# Multistate Redox-Switchable Ion Transport Using Chalcogen-Bonding Anionophores

**DOI:** 10.1021/jacs.2c12892

**Published:** 2023-01-18

**Authors:** Andrew Docker, Toby G. Johnson, Heike Kuhn, Zongyao Zhang, Matthew J. Langton

**Affiliations:** Department of Chemistry, Chemistry Research Laboratory, University of Oxford, Mansfield Road, Oxford OX1 3TA, UK

## Abstract

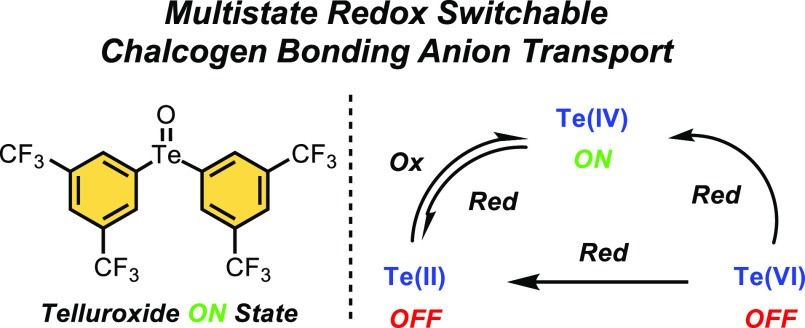

Synthetic supramolecular transmembrane anionophores have emerged as promising anticancer chemotherapeutics. However, key to their targeted application is achieving spatiotemporally controlled activity. Herein, we report a series of chalcogen-bonding diaryl tellurium-based transporters in which their anion binding potency and anionophoric activity are controlled through reversible redox cycling between Te oxidation states. This unprecedented *in situ* reversible multistate switching allows for switching between ON and OFF anion transport and is crucially achieved with biomimetic chemical redox couples.

## Introduction

The precise regulation of charged species in biological systems underpins many of the processes required for life, and as a consequence, ionic imbalances can severely compromise physiological function. Supramolecular ionophores that function as mobile carriers for ions have garnered significant interest as potential therapeutics for diseases associated with ion misregulation,^1−3^ such as cystic fibrosis and Bartter syndrome, or as novel cancer treatment strategies to trigger tumor cell apoptosis.^4−7^ Indeed, in the context of anion transport, there now exists a vast library of predominantly hydrogen bonding (HB)^8−18^ synthetic anion receptors, possessing a wide range of donor motifs and topologies, and capable of facilitating membrane transport for a range of biologically relevant anions. Many of the fundamental factors that govern anionophore transport behavior including activity,^19−23^ anion selectivity,^24,25^ and membrane deliverability,^26^ including in chalcogen bonding systems,^27−30^ have now been established. However, from a clinical perspective, it is highly desirable to develop anion transporter systems that exhibit controllable behavior^31^ (i.e., stimuli-responsive activity as a means of selectively targeting specific tissue). To this end, photoswitchable systems have demonstrated considerable promise, wherein modulation of anion binding behavior by light-induced isomerization in mobile carrier and membrane-anchored systems^32−38^ can effectively switch ON or OFF anion transport. However, another potential strategy toward achieving spatiotemporally controlled anion transport is developing anionophores that are responsive to the target cell’s intrinsic physiological environment.^39^ Considering that the rapid proliferation of cancer cells is typically accompanied by a considerable increase in both intracellular reactive oxygen species (ROS)^40,41^ and glutathione (GSH)^42,43^ concentrations, which comprise some of the major oxidative and reductive relay systems in cell physiology, it is conceivable that designing systems capable of operating in the cell’s *specific redox window* via *reversible* redox activation could be a powerful strategy in the design of targeted anionophore-based chemotherapies. However, the scarce examples of redox-controlled anion transporters reported to date typically rely on *irreversible* chemical modifications of the anion binding motif by redox stimuli, namely, reductive demethylation or metal decomplexation.^44−49^

Chalcogen bonding (ChB), the attractive non-covalent interaction between a group 16 atom and a Lewis base, has recently come to the fore in the context of solution-phase anion recognition, not only frequently exhibiting enhanced affinities but also contrasting selectivity profiles compared to more traditionally employed interactions such as HB.^50−52^ It has recently been demonstrated that the chalcogen-centered Lewis acidity and therefore anion binding potency of ChB donors are highly sensitive to their local electronic environments and are in fact highly tunable through substituent variation,^53^ co-bound cation recognition,^54^ and electrochemical switching.^55^ Indeed, in the context of developing switchable receptors for anion recognition purposes, this unique characteristic and a wide range of reversibly accessible oxidation states for organochalcogen derivatives provide a unique platform for redox-responsive multistate anion binding systems.

**Figure 1:**
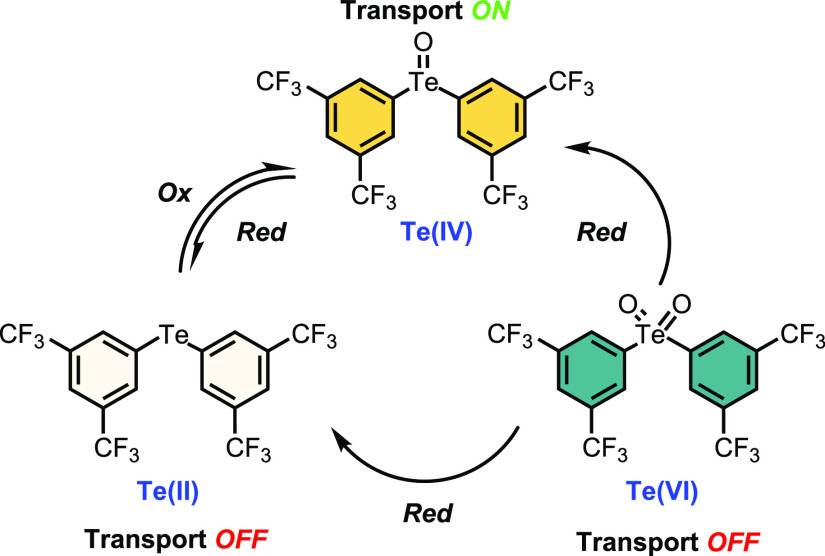
Schematic representation of the redox-addressable multistate anion transport system with **1·Te^2CF3^**.

Herein, we report the first example of multistate redox-switchable transmembrane anion transport. Exploiting a series of diaryl ChB receptor systems, we demonstrate that their transmembrane anion transport capabilities are strongly influenced by the oxidation state of the chalcogen center, corresponding to multiple ON and OFF states. Importantly, by employing either reduction or oxidation reactions that transition between Te(VI), Te(IV), and Te(II) states, it is possible to switch reversibly between ON and OFF transport states *in situ* in the membrane (Figure 1).

## Results and Discussion

### Receptor Design

To identify potential ChB donor systems capable of redox-switchable anion transport, two main criteria had to be met. First, upon transitioning between the oxidized and reduced states, the ChB donor anion binding potency has to be sufficiently modulated as to translate to differing anionophore performance. Second, the associated redox processes must not compromise the chemical integrity of the system, thus facilitating *reversible* cycling between the states.

We therefore sought to exploit a diaryl (Ar = Ph, 3,5-bis(trifluoromethyl)phenyl) chalcogen (Ch = Se and Te) Ar_2_Ch parent scaffold possessing a Ch(II) center,^56^ wherein successive oxidation reactions could provide access to the corresponding Ar_2_ChO and Ar_2_ChO_2_ species, possessing Ch(IV) and Ch(VI) chalcogen centers, respectively. It was envisaged that in addition to the expected increase in chalcogen-centered electrophilicity by the integration of electron-withdrawing groups to the aryl substituents, the increase in the formal oxidation state of Ar_2_ChO relative to Ar_2_Ch will dramatically increase the ChB donor potency and therefore anion transport capabilities. It was also expected that in the case of the most highly oxidized species, Ar_2_ChO_2_, and despite the ostensible increase in chalcogen electrophilicity, the inaccessibility of the sigma-hole at the Ch center would preclude its ability to bind and therefore transport anions. Importantly, cycling between these tellurium species through oxidative or reductive processes is well documented with high levels of chemical fidelity and reversibility.

### Synthesis

**Scheme 1:**
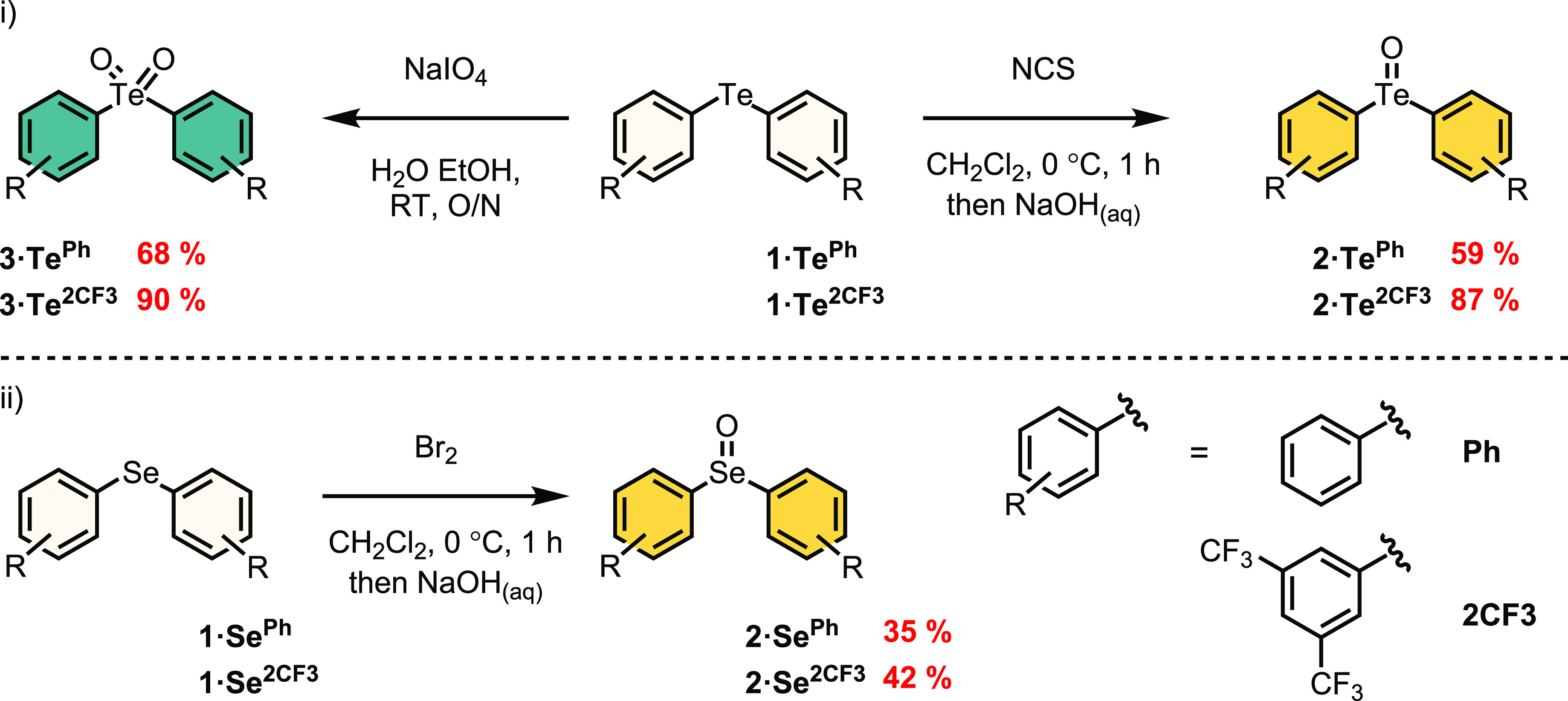
Synthetic Route from (i) the Tellurides **1·Te^Ph^** and **1·Te^2CF3^** to the Telluroxides **2·Te^2CF3^** and **2·Te^2CF3^** and Tellurones **3·Te^Ph^ and 3·Te^2CF3^** and (ii) the Selenides **1·Se^Ph^** and **1·Se^2CF3^** to the Selenoxides **2·Se^2CF3^** and **2·Se^2CF3^**

The requisite parent diaryl chalcogenides **1·Se^Ph^**, **1·Te^Ph^**, **1·Se^2CF3^**, and **1·Te^2CF3^** were prepared according to literature procedures or modified versions thereof (Scheme 1). Access to the telluroxides **2·Te^Ph^** and **2·Te^2CF3^** was achieved via an *N*-chlorosuccinimide-mediated oxidation procedure of the corresponding telluride to form the chloro-telluronium species, which was subsequently hydrolyzed by treatment with NaOH_(aq)_ to give the telluroxides in yields of 59 and 87%, respectively.^57^ Access to the Te(VI) tellurone derivatives **3·Te^2CF3^** and **3·Te^Ph^** was achieved through treatment of the corresponding telluride with NaIO_4_ in EtOH:H_2_O mixtures in excellent yield.^58^ The pronounced resistance of selenium to access higher oxidation states, relative to tellurium analogues, necessitated the use of stronger oxidizing agents to obtain the selenoxides.

Accordingly, elemental bromine was used to generate the corresponding diorgano selenium(IV) dihalides (Ar_2_SeBr_2_), which were hydrolyzed under basic conditions to afford the selenoxides **2·Se^Ph^** and **2·Se^2CF3^** in 35 and 42%, respectively. Interestingly, access to the corresponding Ar_2_SeO_2_ selenones proved considerably more challenging, wherein treatment of the parent selenides with NaIO_4_ resulted in either very poor conversion in the case of the phenyl-substituted derivative **3·Se^Ph^** or no conversion, even after elevated reaction temperatures and prolonged reaction time, in the case of **3·Se^2CF3^**. This is presumably a consequence of the electron-withdrawing bis(trifluoromethyl)aryl unit further increasing resistance to oxidation. All novel compounds were characterized by ^1^H, ^13^C, ^77^Se, and ^125^Te NMR spectroscopy, where appropriate, and high-resolution mass spectrometry (see the Supporting Information for full synthetic procedures and characterization).

### Solid-State Structure Determination

**Figure 2:**
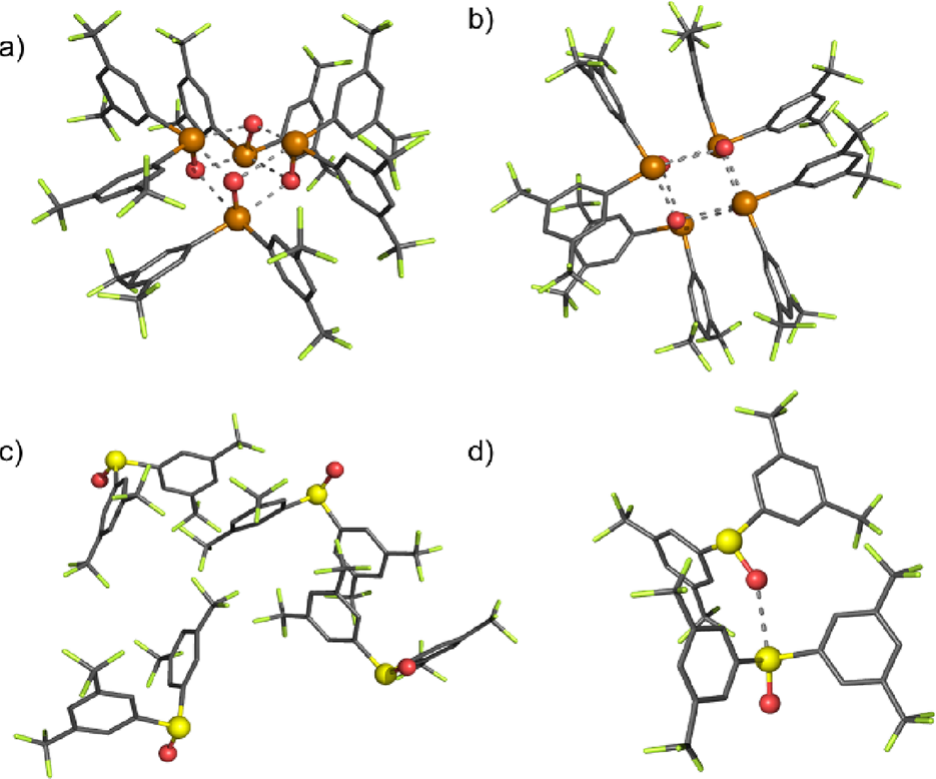
X-ray crystal structure of **2·Te^2CF3^**: (a) side view (b) top view. Crystal structures of **2·Se^2CF3^** showing (c) C–F···π and π··· π interactions and (d) Se···O interactions.

Insight into the differing potency of the tellurium and selenium ChB donors was provided by solid-state characterization of **2·Te^2CF3^** and **2·Se^2CF3^** (Figure 2). Crystals of **2·Te^2CF3^** and **2·Se^2CF3^** suitable for X-ray structural analysis were grown by slow diffusion of diethyl ether into acetonitrile solutions. The determined structure of **2·Te^2CF3^** reveals a highly ordered square-like tetrameric arrangement of alternatingly oriented **2·Te^2CF3^** molecules wherein each Te center exhibits very short (in the range of 2.415–2.987 Å) bifurcated Te···O interactions with two other molecules. Of particular note is the significant 72 and 74% contraction in the van der Waals radii for Te1···O1 and Te1···O3, indicative of strong intermolecular ChB sigma-hole interactions in the solid state. In contrast, the selenium congener **2·Se^2CF3^** possesses a much less well-organized molecular arrangement wherein C–F···π and π··· π interactions appear to be the principal interactions governing the adoption of a chain-like structure, with only a single long Se1···O1 contact observed.

### Chloride Anion Recognition Studies

To assess the chloride recognition properties of the proposed ChB transporters, ^1^H NMR titration experiments were conducted in CD_3_CN solution with tetrabutylammonium chloride. Addition of increasing equivalents of Cl^–^ to solutions of either **2·Te^2CF3^** or **2·Te^Ph^** resulted in a downfield perturbation of the aryl proton resonance *ortho* substituted to the tellurium center, indicative of an anion recognition event occurring via the ChB Te···Cl^–^ interaction (Figures S10 and S11). Analysis of the generated binding isotherm (Figure S13) using Bindfit^54^ determined 1:1 host:guest association constants (*K*_a_) of 935 and 197 M^–1^ for **2·Te^2CF3^** and **2·Te^Ph^**, respectively, which is consistent with the ChB donor strength being enhanced by the presence of inductively activating electron-withdrawing groups. The lower oxidation state tellurides, **1·Te^2CF3^** or **1·Te^Ph^**, exhibit no measurable chloride binding affinities. In contrast, neither the selenoxides **2·Se^2CF3^** and **2·Se^Ph^** nor the selenides **1·Se^2CF3^** and **1·Se^Ph^** exhibit measurable chloride affinity, which is understandable when considering that the strength of sigma-hole donors typically correlates with element polarizability.^a^

### Redox Switching Studies

**Scheme 2:**
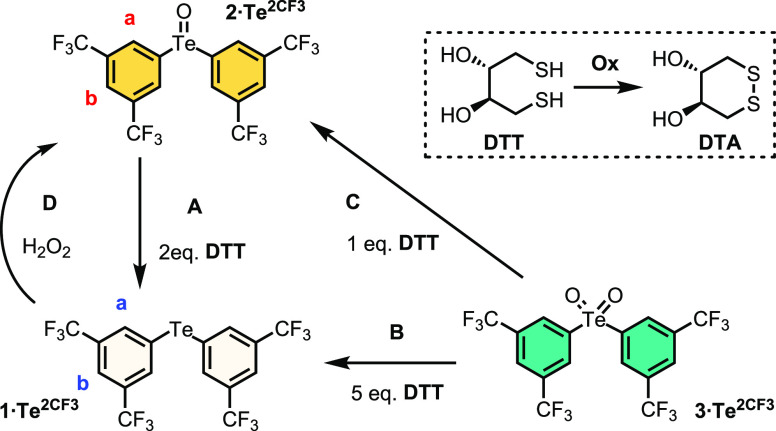
Summarized Redox Transformations between **1·Te^2CF3^**, **2·Te^2CF3^**, and **3·Te^2CF3^**, mediated by DTT or peroxide Inset showing the DTT to DTA redox couple.

To confirm the chemical accessibility of the desired oxidation states, preliminary oxidation–reduction studies were conducted in CD_3_CN, summarized in Scheme 2. First, it was confirmed that organically soluble glutathione mimics (i.e., dithiothreitol (DTT)) are capable of reducing the telluroxides and tellurones to the corresponding tellurides, processes A and B, respectively, through the thiol–disulfide redox couple (DTT to dithiane DTA), and that a sequential transition through the corresponding Te states, Te(VI) → Te(IV) → Te(II), is achievable by careful stoichiometric control of the thiol concentration, processes C followed by A (Figure S14). The oxidative process D of Te(II) → Te(IV) was also shown to be possible by treatment with H_2_O_2_. Interestingly, while the reduction of the selenoxides to the selenides was also possible by treatment with DTT, the corresponding reverse process (oxidation from selenide to selenoxide) was not observed after treatment with H_2_O_2_, which again presumably reflects the increased resistance of selenium, relative to tellurium, to oxidation.

### Transmembrane Anion Transport Activity

Attention was then directed to investigating the chloride anion transport capabilities of the selenium- and tellurium-based receptor series. The anion transport activities were determined in 1-palmitoyl-2-oleoyl-*sn*-glycero-3-phosphocholine large unilamellar vesicles (POPC LUVs, lipid concentration 31 μM), loaded with 8-hydroxypyrene-1,3,6-trisulfonate (HPTS) in NaCl solution buffered to pH 7.0 with HEPES. A pH gradient was applied across the membrane by addition of a base pulse, followed by addition of the carrier as a DMSO solution (<0.5% v/v). The ability of the anionophore to dissipate the pH gradient by transmembrane ion transport (Cl^–^/OH^–^ antiport or H^+^/Cl^–^ symport) was determined by recording the change in the HPTS emission, *I*_rel_ (λ_em_ = 510 nm), with time following excitation at λ_ex_ = 405/465 nm. The addition of detergent (Triton X-100) facilitated calibration of the emission intensity.

**Figure 3:**
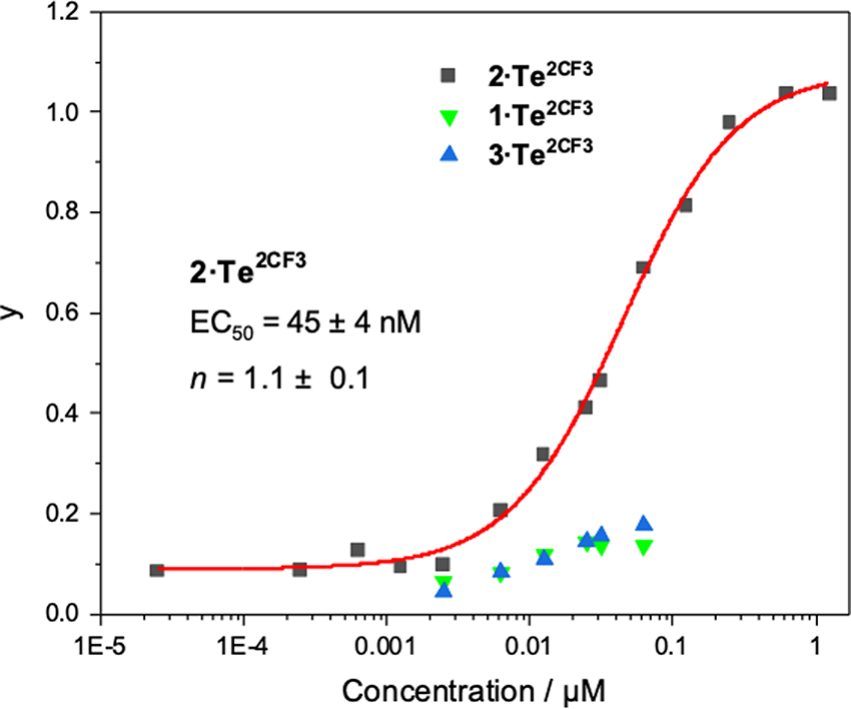
Hill plot analysis of the relative anion transport activities, *y*, of **2·Te^2CF3^** in POPC LUVs and the corresponding activities of **1·Te^2CF3^** and **3·Te^2CF3^** across a range of concentrations.

This assay was used to determine the concentration dependence of the transport activity of each receptor. The transport data for the Te receptor series functionalized with the electron-withdrawing 3,5-bis(trifluoromethyl)aryl groups, **1·Te^2CF3^**, **2·Te^2CF3^**, and **3·Te^2CF3^**, is shown in Figure 3 (see the Supporting Information for data for the remaining compounds). The fractional activities *y* (relative intensities immediately prior to vesicle lysis) were plotted as a function of concentration and the dose–response curves fitted to the Hill equation. This describes the dependence of the anion transport activity (reflected in *I*_rel_) on the *n*th power of the carrier concentration, facilitating comparison of relative activities through an effective concentration value (EC_50_) required to reach 50% activity. The *n*th power, or the Hill coefficient, may be interpreted as the stoichiometry of the receptor:anion supramolecular complex implicated in facilitated transport.^59^

Of the series, the Te(IV) telluroxide **2·Te^2CF3^** demonstrated considerable anion transport activity with an EC_50_ = 45 nM, particularly impressive for such a simple electroneutral receptor relying on a single donor atom. A determined *n* value of ∼1 for **2·Te^2CF3^** indicates that anion transport operates through a 1:1 **2·Te^2CF3^**:chloride complex, presumably similar to that observed from ^1^H NMR anion titration experiments. Importantly, transport was not detected when chloride was replaced with gluconate, a larger hydrophilic anion, which is consistent with the mobile carrier being unable to overcome the significant dehydration enthalpy required for a OH^–^/gluconate antiport mechanism of transport (Figure S3). Anion transport by **2·Te^2CF3^** in the lipid gel phase of dipalmitoylphosphatidylcholine (DPPC) lipids at 25 °C was inhibited, and restored when heated to 45 °C, above the gel–liquid phase transition temperature (*T*_c_ = 41 °C) (Figure S4). This behavior is consistent with the proposed mobile carrier mechanism, in which the mobility through the lipid bilayer is dramatically reduced in the gel phase, as opposed to a channel-type mechanism that would likely be lipid phase independent. The activities of **1·Te^2CF3^**, **2·Te^2CF3^**, and **3·Te^2CF3^** when pre-incorporated into vesicles during preparation were comparable to those obtained via post-incorporation (Figure S5), which rules out aggregation^27^ effects or variable deliverability^13,26,60^ being responsible for the observed redox switching of activity.

The chloride over hydroxide selectivity of **2·Te^2CF3^** was also investigated through an “NMDG” assay, established by Gale, Davis, and co-workers,^25,61^ in which NaCl is replaced by *N*-methyl-d-glucamine chloride (NMDGCl) in the internal and external buffer, and the sodium hydroxide base pulse exchanged with NMDG (5 mM) (Figure S6). The ratio of the determined EC_50_ values in the presence and absence of gramicidin was ∼2, indicating modest chloride over hydroxide selectivity.

With the transport behavior of **2·Te^2CF3^** characterized, attention turned to investigating the transport capabilities of the Te(II) telluride and Te(VI) tellurone analogues, **1·Te^2CF3^** and **3·Te^2CF3^**, respectively. In stark contrast to **2·Te^2CF3^**, both **1·Te^2CF3^** and **3·Te^2CF3^** exhibit negligible activity over a range of concentrations, which includes the EC_50_ of **2·Te^2CF3^** (Figure 3). Interestingly, the phenyl-appended Te-based analogues exhibited a similar trend, where the **2·Te^Ph^** exhibits moderate activity (EC_50_ = 340 nM), attenuated with respect to **2·Te^2CF3^** (Figure S2). This may be rationalized on the basis of reduced chloride affinity, while the **1·Te^Ph^** and **3·Te^Ph^** exhibit no measurable activity. In accordance with the lack of measurable chloride binding affinities observed by ^1^H NMR of the selenium derivative series, **1·Se^Ph^**, **2·Se^Ph^**, **2·Se^2CF3^**, and **2·Se^2CF3^** exhibited no transmembrane chloride transport capabilities by the aforementioned HPTS assay.

### Redox-Switchable Anion Transport Activity

Encouraged by the observation that **2·Te^2CF3^** exhibits effective anion transport capability in contrast to the inactive species **1·Te^2CF3^** and **3·Te^2CF3^** and is related by reversible oxidative or reductive processes, efforts were undertaken to access these states in an *in situ* fashion and thereby achieve redox-controlled anion transport in vesicles.

#### ON–OFF Switching of Ion Transport

**Figure 4:**
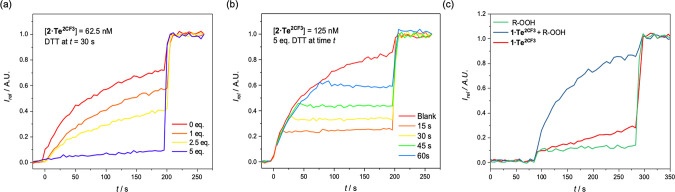
Anion transport in the HPTS assay. Data for **2·Te^2CF3^** is shown. Change in ratiometric emission (λ_em_ = 510 nm; λ_ex1_ = 405 nm, λ_ex2_ = 460 nm) upon (a) addition of **2·Te^2CF3^** after incubation with varying equivalents of DTT prior to base pulse addition at *t* = 0 s. (b) After the addition of 5 equiv of DTT at a given time *t*. (c) OFF–ON studies for **1·Te^2CF3^(**62.5 nM) with an organic peroxide R-OOH (cumene hydroperoxide, 12.5 μM, incubated for 60 min prior to base pulse addition).

First, we investigated the possibility of exploiting the activity of the Te(IV) system, while its reduced Te(II) form exhibits no significant activity. To this end, we sought to achieve an *in situ* generation of inactive **1·Te^2CF3^** from active **2·Te^2CF3^** via a thiol–disulfide redox couple, which would correspond to switching between ON–OFF anion transport states. Specifically, **2·Te^2CF3^** was added to HPTS-loaded vesicles, followed by the addition of either 1, 2.5, or 5 equiv of DTT, and incubated for 30 s, after which time a base pulse was added to initiate transport (Figure 4a). The measured transport activity decreased with increasing equivalents of DTT, concordant with a decreasing concentration of the “active” **2·Te^2CF3^** species, and to the extent that samples incubated with 5 equiv of DTT exhibit no measurable transport. This corresponds to complete reduction of the telluroxide to the inactive telluride **1·Te^2CF3^**. Similar results were obtained for the less active **2·Te^Ph^**, where transport could also be switched off by DTT addition (Figure S7). Motivated by this, we sought to investigate whether this *in situ* reduction could be exploited in a “real-time” fashion, in which transport could be effectively halted by the addition of DTT to the vesicles during a “live” transport experiment. To this end, we conducted the conventional HPTS transport assay with carrier **2·Te^2CF3^**, during which at a given time *t*, 5 equiv of DTT was added (Figure 4b). Pleasingly, upon the addition of the reductant, an almost immediate halt in transport activity was observed, indicating that the *in situ* reduction of **2·Te^2CF3^** → **1·Te^2CF3^** does indeed translate to ability to switch from ON → OFF transport states with impressive levels of temporal control.

#### OFF–ON Switching

Given the ability to transition from ON → OFF transport states in an *in situ* fashion via a reductive transformation of **2·Te^2CF3^** to **1·Te^2CF3^**, we explored the possibility of performing the reverse process, namely, *in situ* oxidation of **1·Te^2CF3^** to **2·Te^2CF3^**, corresponding to switching on transport (OFF → ON). In a similar manner to the reductive incubation experiments above, an aliquot of **1·Te^2CF3^** as a DMSO solution was added to HPTS-loaded vesicles, after which an aqueous solution of H_2_O_2_ (12.5 μM) was added and left to incubate for 45 min. Subsequent addition of a NaOH solution established the requisite pH gradient to initiate transport, and appreciable anion transport activity was observed (Figure S8), while in the absence of oxidant **1·Te^2CF3^** remained inactive. It is interesting to note that despite the addition of a considerable excess of oxidant (100-fold excess relative to **1·Te^2CF3^**) and the longer incubation times relative to those used for the reduction process mediated by DTT, only ∼35% of the hypothetical maximum activity is observed (i.e., the expected transport if **1·Te^2CF3^** was fully converted to the Te(IV) state at the used concentration). This is presumably due to either the slower kinetics of the oxidation reaction relative to the DTT-mediated reduction process. In contrast, however, incubation of **1·Te^2CF3^** with a more hydrophobic peroxide based oxidant, cumene hydroperoxide, resulted in enhanced switching from **1·Te^2CF3^** → **2·Te^2CF3^** and near quantitative restoration of activity (Figure 4C). This ability to perform an *in situ* oxidation reaction of **1·Te^2CF3^** to **2·Te^2CF3^** corresponds to a switch of OFF → ON in anion transport, mediated by a redox couple.

#### OFF–ON–OFF Switching

**Figure 5:**
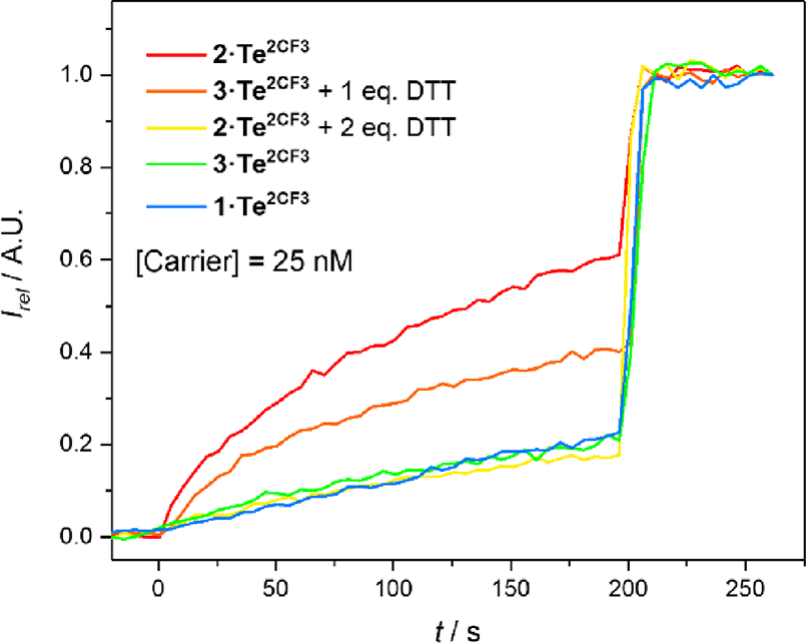
OFF–ON–OFF switching experiments in which **3·Te^2CF3^** is incubated with various equivalents of DTT.

Motivated by the success of the previous switching experiments, we were interested in whether, through careful stoichiometric control of the DTT reductant, the inactive Te(VI) tellurone could be reduced to the active Te(IV) telluroxide while ambitiously avoiding complete reduction to the inactive Te(II) telluride. To probe this concept, we first performed incubation experiments in which **3·Te^2CF3^** was added to HPTS-loaded vesicles together with either 1 or 5 equiv of DTT and left to incubate for 30 s, after which a base pulse was added, and the transport activity determined. When 5 equiv of DTT was added, transport equivalent to that of the background activity of **1·Te^2CF3^** at this concentration was observed, implying complete reduction of **3·Te^2CF3^** to the inactive **1·Te^2CF3^**. In contrast, incubation of equimolar quantities of **3·Te^2CF3^** and DTT for 30 s prior to base pulse addition resulted in considerable activity, implying conversion to the active Te(IV) species (Figure 5). As observed in the OFF–ON switching studies, the maximum hypothetical activity (corresponding to quantitative conversion of **3·Te^2CF3^** to **2·Te^2CF3^**) is not observed and the activity is ∼50% of what is anticipated for **2·Te^2CF3^** at this concentration, which is most likely attributable to generation of a distribution of redox states comprising the active **2·Te^2CF3^**, the inactive fully reduced **1·Te^2CF3^**, and unreacted **3·Te^2CF3^**.

**Figure 6:**
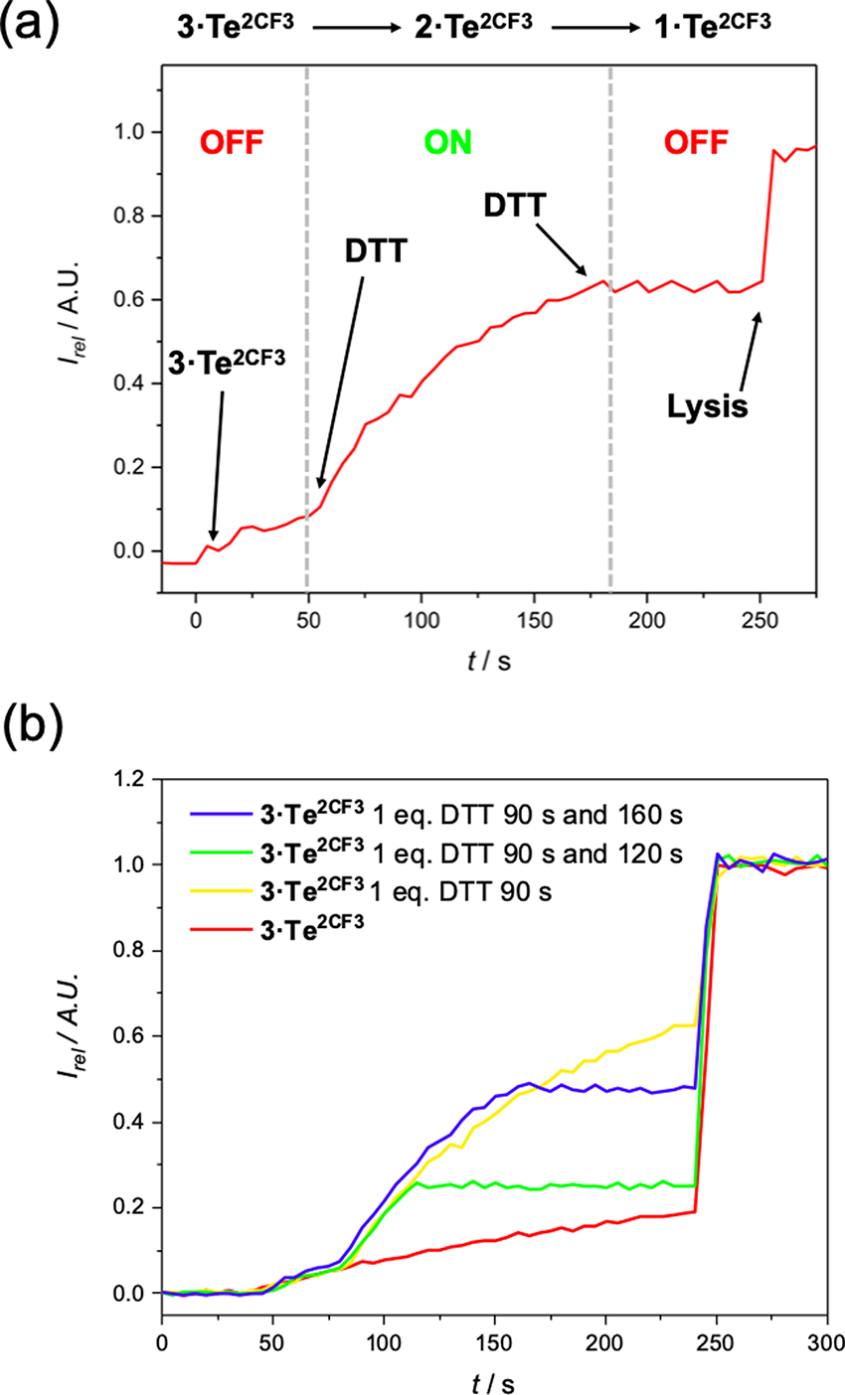
(a) Real-time *in situ* OFF–ON–OFF switching experiments wherein **3·Te^2CF3^** is serially reduced with DTT additions. (b) OFF–ON–OFF switching at various time intervals. Initial [**3·Te^2CF3^** ] **=** 62.5 nM.

Given the rapid kinetics associated with the reduction process mediated by DTT, we envisaged that it would also be possible to access the following reaction sequence via serial reduction reactions, **3·Te^2CF3^** → **2·Te^2CF3^** → **1·Te^2CF3^**, which would correspond to OFF → ON → OFF states of anion transport. With this in mind, we conducted the conventional HPTS assay with **3·Te^2CF3^**, which was allowed to run for 50 s, during which time transport corresponding to the background activity was observed (OFF state). At 50 s, a DMSO solution of DTT was added, at a concentration corresponding to 1 equiv relative to **3·Te^2CF3^** (Figure 6). Immediately after the addition, the profile of the fluorescence intensity change was perturbed, corresponding to the generation of a more active species (i.e., **2·Te^2CF3^**) and a switch ON of activity. After an additional 125 s had elapsed, a second portion of 2.5 equiv of DTT was added, which resulted in a complete switch OFF of activity, corresponding to the generation of the inactive Te(II) (i.e., **1·Te^2CF3^**). Pleasingly, this ability to conduct serial *in situ* reduction on this telluone–telluroxide–telluride redox relay system in the lipid membrane bilayer means that we can achieve unprecedented time-resolved OFF–ON–OFF anion transport. Indeed, it is noteworthy that while redox-controlled strategies remain very rare in the context of stimuli-responsive ion transport, the few reported examples rely on irreversible chemical modifications of pro-anionophores, corresponding to unidirectional and “single use” OFF → ON switching.^44−49^ Our methodology not only constitutes the first example bidirectional switching (i.e., OFF → ON and ON → OFF) but also presents a hitherto unexplored opportunity for multistate switching (OFF → ON → OFF).

## Conclusions

In conclusion, we have developed a strategy to achieve multistate redox-responsive transmembrane anion transport. Exploiting a diaryl organotellurium derivative, reversible oxidation and reduction transformations occurring at the Te center serve to dramatically modulate the ChB donor anion binding potency and therefore anionophore activity. Redox transitioning between either Te(IV) → Te(II) (and vice versa) or Te(VI) → Te(IV) demonstrates that it is possible to switch between the following states of transmembrane anion transport: ON → OFF and OFF → ON. Furthermore, we have also demonstrated for the first time multistate addressable anion transport in an OFF → ON → OFF redox relay, through a serial reductive methodology operating through Te(VI) → Te(IV) → Te(II) redox states. Perhaps most importantly, the fact that these redox-mediated switching pathways are achieved through either a reduction with a thiol–disulfide redox couple, an analogue for the biological GSH/GSSG cycle, or oxidation with peroxide, which is a reactive oxygen species produced during cell metabolism, serves to exemplify that this strategy of redox-responsive ChB-mediated anionophores poses genuine therapeutic promise of exploiting a physiological redox window as a means of achieving targeted activity.

## Supporting Information Available

Experimental procedures, characterization of new compounds, NMR titrations, redox switching, and anion transport studies (PDF)

## Footnotes

a While it was not possible to determine the chloride binding properties of **3·Te^2CF3^** by ^1^H NMR titrations, due to the characteristically broad proton signals of this compound, the inactivity of **3·Te^2CF3^** in the chloride transport HPTS assay is strong evidence of minimal chloride binding affinity.
