# Supplementary material for: Multistate Redox-Switchable Ion Transport Using Chalcogen-Bonding Anionophores
Source: J Am Chem Soc. 2023 Jan 18;145(4):2661–8. doi: 10.1021/jacs.2c12892 (PMC9896566; doi:10.1021/jacs.2c12892)
Supplement: Supplementary file 1 — ja2c12892_si_001.pdf [file ja2c12892_si_001.pdf]

Supporting information for

Multistate Redox Switchable Ion Transport Using Chalcogen Bonding Anionophores

Andrew Docker, Toby G. Johnson, Heike Kuhn, Zongyao Zhang and Matthew J. Langton*[a]

[a]Dr. A. Docker, H. Kuhn, T.G. Johnson, Z. Zhang, Prof. M. J. Langton, Department of Chemistry University of Oxford, Chemistry Research Laboratory, Mansfield Road, Oxford OX1 3TA (UK)

E-mail: matthew.langton@chem.ox.ac.uk

Contents

Materials and methods 2
Anion transport experiments 3
  HPTS assay data for transporters 4
  Gluconate Assay 5
  Membrane fluidity studies 6
  NMDG Assay 7
  ON to OFF Switching for $1\cdot Te^{Ph}$ 8
  OFF to ON switching 9
  Pre-incorporation studie 6
^{1}H NMR Titration Experiments 11
NMR Switching Studies 13
Synthesis and Characterisation 14
Single Crystal X-ray diffraction experiments 31
References 34

Materials and methods

All reagents and solvents were purchased from commercial sources and used without further purification. Lipids were purchased from Avanti polar lipids and used without further purification. Where necessary, solvents were dried by passing through an MBraun MPSP-800 column and degassed with nitrogen. Column chromatography was carried out on Merck® silica gel 60 under a positive pressure of nitrogen. Where mixtures of solvents were used, ratios are reported by volume. NMR spectra were recorded on a Bruker AVIII 400, Bruker AVII 500 (with He cryoprobe) and Bruker AVIIIHD 500 spectrometers. Chemical shifts are reported as δ values in ppm. Mass spectra were carried out on an Agilent 6120 bench-top single quadrupole, a Waters LCT Premier XE benchtop (oa-TOF) and a Thermo Exactive HighResolution Orbitrap FTMS spectrometer. Fluorescence spectroscopic data were recorded using a Horiba Duetta fluorescence spectrophotometer, equipped with Peltier temperature controller and stirrer. Experiments were conducted at 25°C unless otherwise stated. Vesicles were prepared as described below using Avestin "LiposoFast" extruder apparatus, equipped with polycarbonate membranes with 200 nm pores. GPC purification of vesicles was carried out using GE Healthcare PD-10 desalting columns prepacked with Sephadex G-25 medium

Abbreviations:
$CDCl_3$: deuterated chloroform; ChB: Chalcogen Bonding; CuAAC: Copper(I)-catalyzed azide-alkyne cycloaddition; DCM: Dichloromethane; DMSO: Dimethylsulfoxide; DPPC: 1,2-dipalmitoyl-sn-glycero-3-phosphocholine; EC_{50}: Effective concentration; HEPES: N-(2- hydroxyethyl)piperazine-N'-(2-ethanesulfonic acid); HPTS: 8-hydroxy-1,3,6- pyrenetrisulfonate; HRESI MS: High resolution electrospray ionisation mass spectrometry; NaOH: Sodium hydroxide; LUVs: large unilamellar vesicles; MeOH: Methanol; NaCl: Sodium chloride; NMDG-Cl: N-methyl-D-glucamine chloride; POPC: 1-palmitoyl-2-oleoyl-sn-glycero-3-phosphocholine; TBA salt: Tetrabutylammonium salt; THF: Tetrahydrofuran.

Anion transport experiments

Vesicle preparation. A thin film of lipid (1-palmitoyl-2-oleoyl-sn-3-phosphatidylcholine POPC, egg-yolk phosphatidylglycerol EYPG or dipalmitoyl phosphatidylcholine DPPC) was formed by evaporating a chloroform solution under reduced pressure on a rotary evaporator (40 °C) and then under high vacuum for 6 hours. The lipid film was hydrated by vortexing with the prepared buffer (100 mM NaCl, 10 mM HEPES, 1 mM 8-Hydroxypyrene-1,3,6-trisulfonic acid trisodium salt (HPTS), pH 7.0). The lipid suspension was then subjected to 5 freeze-thaw cycles using liquid nitrogen and a water bath (40°C) followed by extrusion 19 times through a polycarbonate membrane (pore size 200 nm). Extravesiclular components were removed by size exclusion chromatography on a Sephadex G-25 column with 100 mM NaCl, 10 mM HEPES, pH 7.0. Final conditions: LUVs (2.5 mM lipid); inside 100 mM NaCl, 10 mM HEPES, 1 mM HPTS, pH 7.0; outside: 100 mM NaCl, 10 mM HEPES, pH 7.0. Vesicles for the sodium gluconate assay were prepared by the same procedure, substituting NaCl for NaGluconate in the buffer solution.

Transport assays with HPTS In a typical experiment, the LUVs containing HPTS (25 μL, final lipid concentration 31.3 μM) were added to buffer (1950 μL of 100 mM NaCl, 10 mM HEPES, pH 7.0) at 25°C under gentle stirring. A pulse of NaOH (20 μL, 0.5 M) was added to initiate the experiment, before the test transporter (various concentrations, in 5 μL DMSO) was added. Detergent (25 μL of Triton X-100 in 7:1 (v/v) H_2O-DMSO) was added at the end of the run after a total of 276 s to calibrate the assay. The fluorescence emission was monitored at λ_{em} = 510 nm (λ_{ex} = 405/460 nm). The fractional fluorescence intensity (I_{rel}) was calculated from equation (S1), where R_t is the fluorescence ratio at time t, R_0 is the fluorescence ratio prior to addition of transporter, and R_d is the fluorescence ratio after the addition of detergent.

$$I_{rel} = \frac{R_t - R_0}{R_d - R_0} \quad \text{(S1)}$$

The fractional fluorescence intensity (I_{rel}) immediately prior to lysis, defined as the fractional activity y, was plotted as a function of the ionophore concentration (x / μM). Hill coefficients (n) and EC_{50} values were calculated by fitting to the Hill equation (S2):

$$y = y_0 + (y_{max} - y_0)\frac{x^n}{EC_{50} + x^n} \quad \text{(S2)}$$

where y_0 is the fractional activity in the absence of transporter, y_{max} is the fractional activity with excess transporter, x is the transporter concentration in the cuvette. Experiments with DPPC lipids were conducted in the same way. For elevated temperature studies, the buffer was equilibrated at 45°C (using the Peltier temperature controller) for 5 minutes prior to initiating the experiment.

Experiments with NMDG-Cl (100 mM) in place of NaCl in both the external and internal buffer was also carried out according to the above procedures, except that the POPC lipid concentration was increased to 0.1 mM and NMDG (20 μL of 0.5 M giving a final concentration 5 mM in the cuvette) was used for the base pulse in place of NaOH. This assay was carried out with and without the addition of the proton channel Gramicidin D (0.1 mol%, added in 4 μL DMSO), which was added 40 s before the base pulse .

HPTS assay data for transporters

In the following figures: Left: change in relative fluorescence intensity over time in the HPTS assay (LUVs (31.3 µM lipid); inside 100 mM NaCl, 10 mM HEPES, 1 mM HPTS, pH 7.0; outside: 100 mM NaCl, 10 mM HEPES, pH 7.0). ***Right:*** dependence of the fractional transport activity *y* in the HPTS assay on the concentration of transporter (black squares) and fitted to the Hill equation (red line).

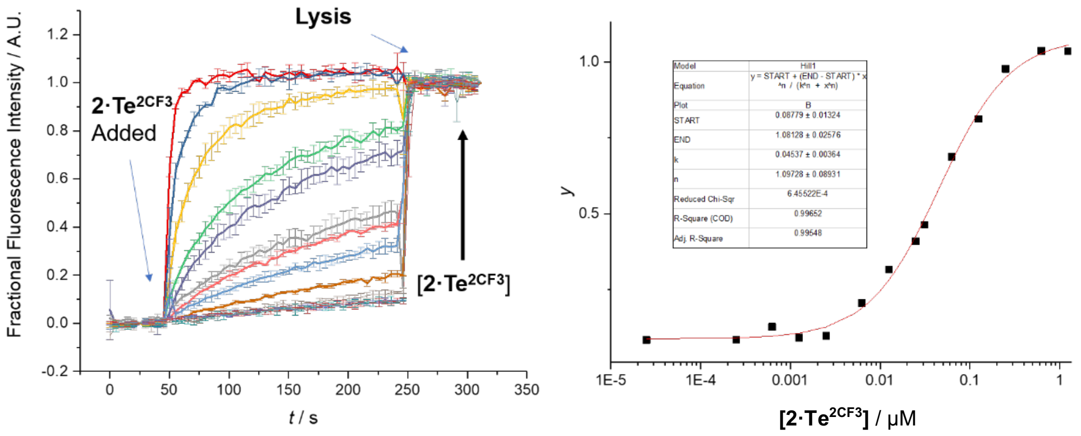

Figure S1. HPTS assay for **2·Te^{2CF3}**.

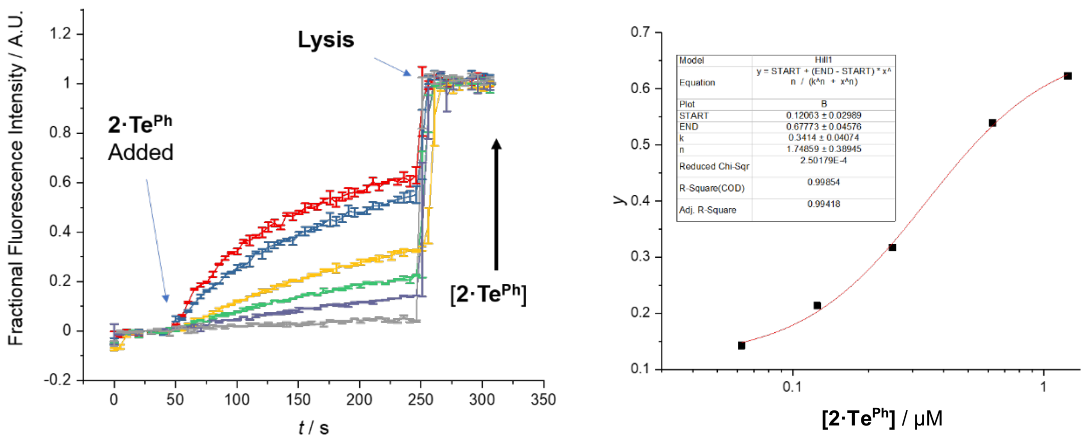

Figure S2. HPTS assay for **2·Te^{Ph}**.

Gluconate Assay

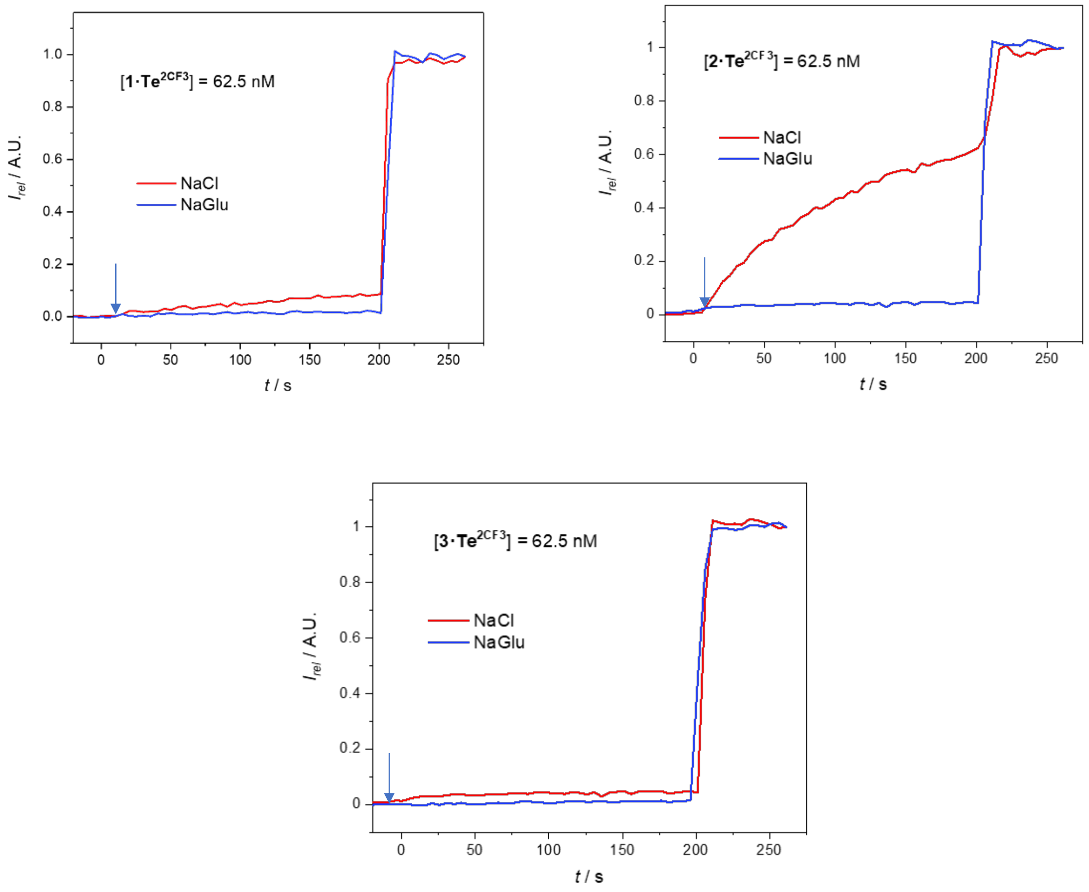

Figure S3. The time-dependent change in fluorescence intensity within the gluconate variation of the HPTS assay. Carriers concentration = 62.5 nM. (a) **1·Te^{2CF3}**, (b) **2·Te^{2CF3}**, (c) **3·Te^{2CF3}** (addition point indicated by arrow). Lysis at 200s using. The decrease in transport with NaGlu for all carriers indicated they do not facilitate sodium cation transport (H+ /Na+ antiport), and must therefore operate via an anion transport mechanism.

Membrane fluidity studies

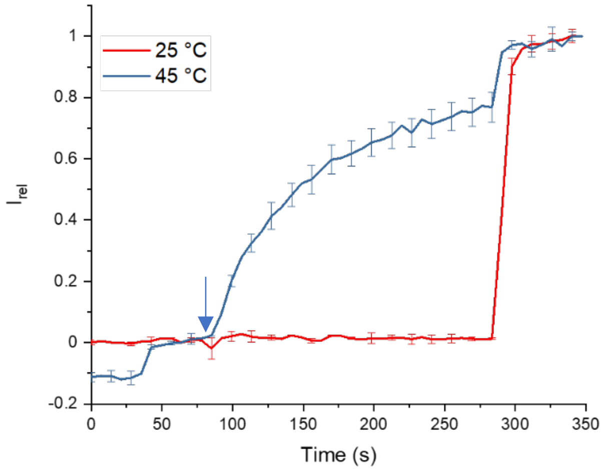

Figure S4. The change in relative fluorescence intensity over time in the HPTS assay utilizing DPPC LUVs at 25°C and 45°C. The temperature of the sample was controlled using a Peltier temperature controller. **2·Te^{2CF3}** was administered (arrow) at a concentration of 62.5 nM. Lack of transport in the gel phase at 25°C, and restoration above the phase transition temperature (41°C) at 45°C, is consistent with a mobile carrier transport mechanism.

Pre-incorporation studies

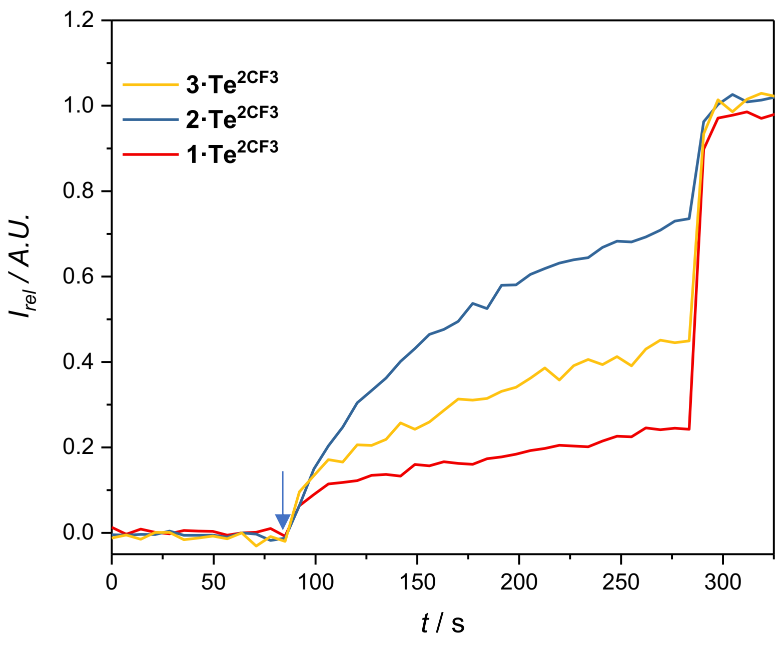

Figure S5. Pre-incorporation studies for **1·Te^{2CF3}**, **2·Te^{2CF3}** and **3·Te^{2CF3}** is shown. Carrier concentration = 31.25 μM (0.2 mol% with respect to POPC). Base pulse addition indicated (arrow).

NMDG Assay

The following figure shows the dependence of the fractional transport activity y in the NMDG assay on the concentration of transporter, and the corresponding fit to the Hill equation (red and blue lines). This assay was carried out with and without the addition of Gramicidin D (0.1 mol%, 4 μL DMSO solution) added 40 s before the NMDG base pulse (20 μL of 0.5 M). Error bars represent 2 s.d.

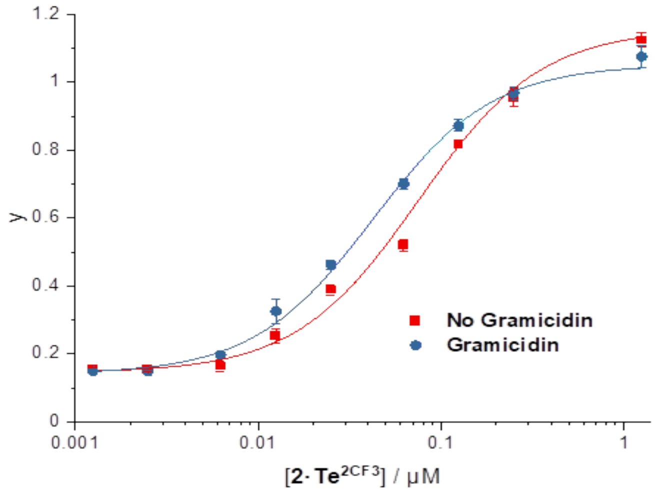

Figure S6. NMDG assay for carrier **2·Te^{2CF3}** with and without gramicidin.

ON to OFF Switching for 2·Te^{Ph}

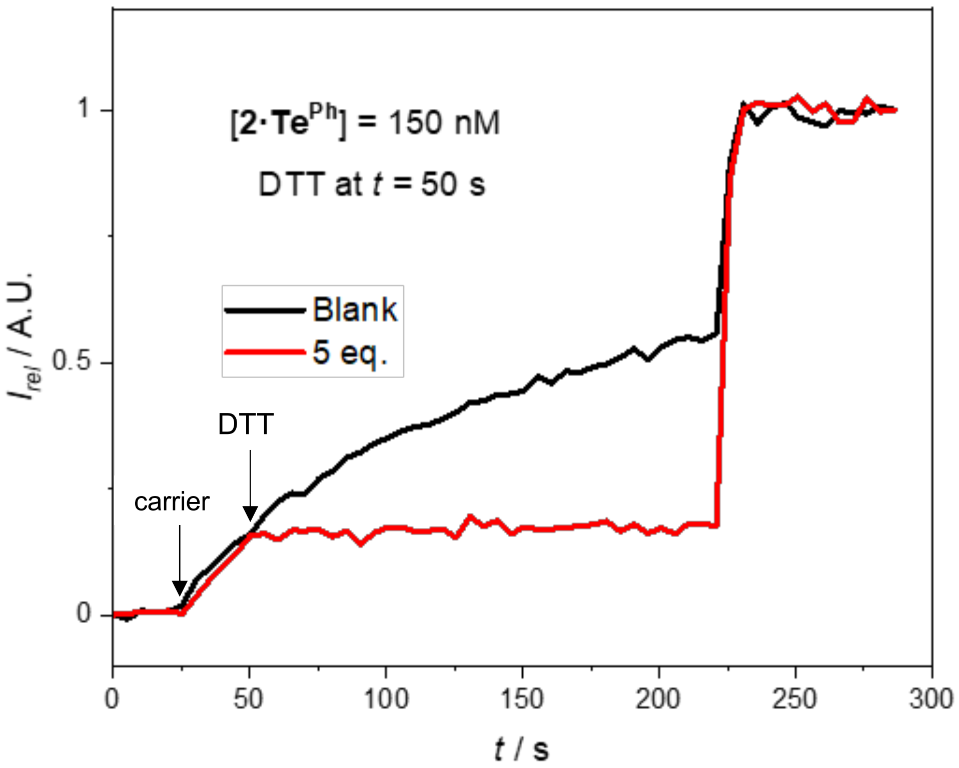

Figure S7. ON-OFF switching in the HPTS assay for **2·Te^{Ph}** is shown. Black line indicates change in fractional intensity after the addition of a DMSO blank and red line indicates change after the addition of 5 equivalents of DTT at 50 s.

OFF to ON switching

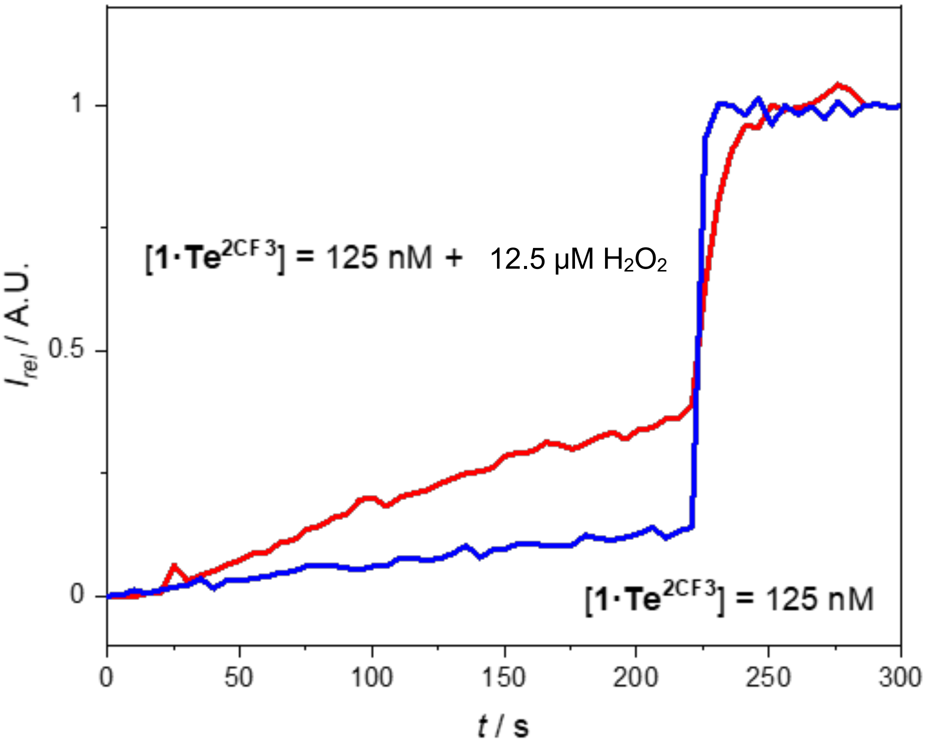

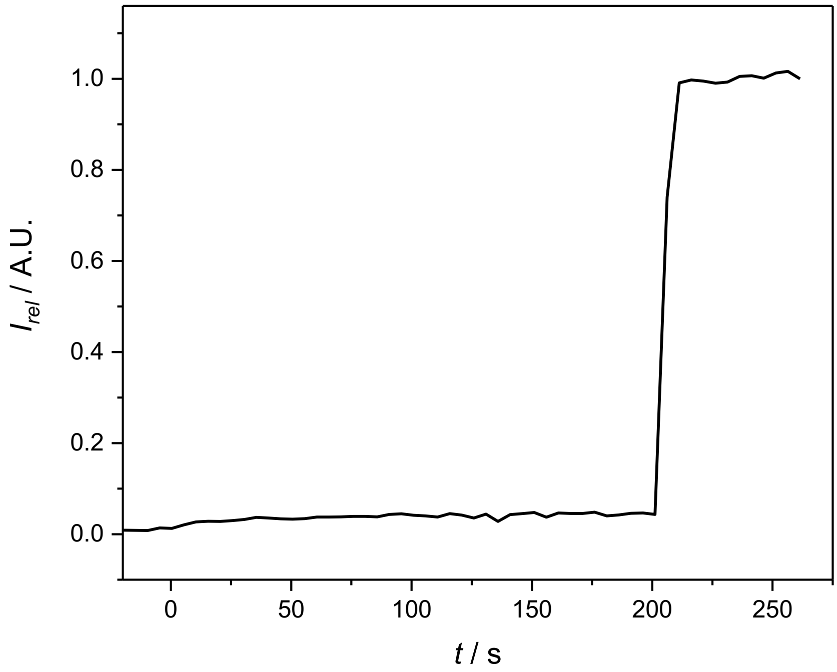

Figure S8. The change in relative fluorescence intensity over time in the HPTS assay. Red line indicates **1·Te^{2CF3}** incubated with 12.5 µM H_2O_2 (100 eq.) and the blue line incubated with a H_2O 'blank' (Top). POPC vesicles incubated with an equivalent addition of H_2O_2 (Bottom).

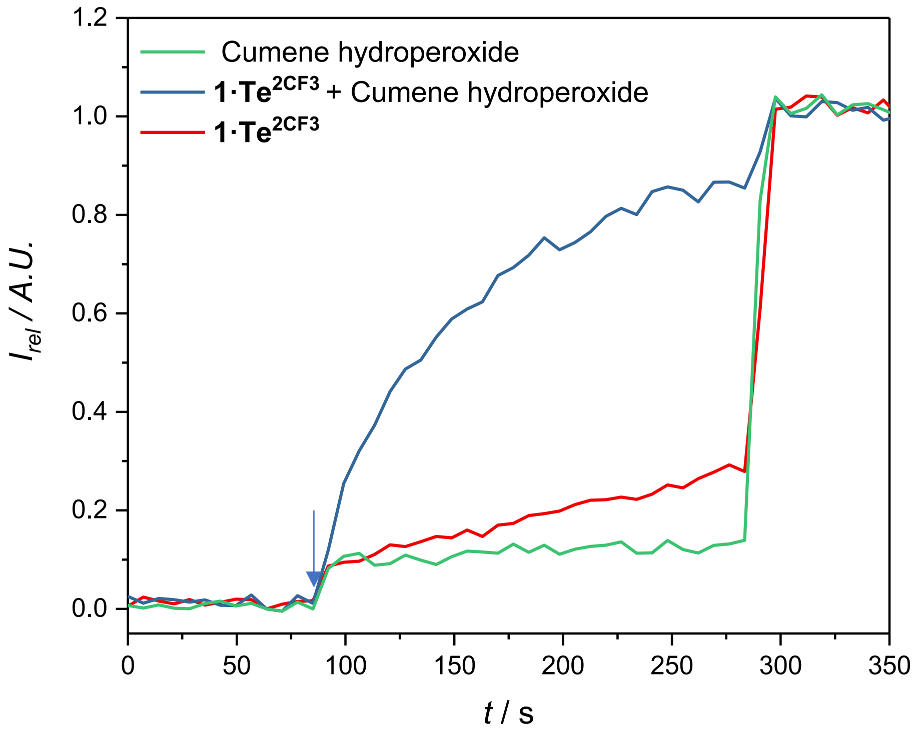

Figure S9. OFF-ON studies for **1·Te^{2CF3}** with cumene hydroperoxide. [Carrier] = 62.5 nM, [Cumene hydroperoxide] = 12.5 μM, incubated for 60 mins prior to base pulse addition (arrow).

^{1}H NMR Titration Experiments

Titration protocol: In a typical ^{1}H NMR anion titration experiment, aliquots of a TBACl in CD_3CN (50 mM) were added to a 1 mM CD_3CN solution of the receptor. Spectra were recorded at 0, 0.2, 0.4, 0.6, 0.8, 1.0, 1.2, 1.4, 1.6, 1.8, 2.0, 2.5, 3.0, 4.0, 5.0, 7.0 and 10 equivalents. The experimental isotherms were fitted to a 1:1 host:guest binding isotherm using Bindfit.

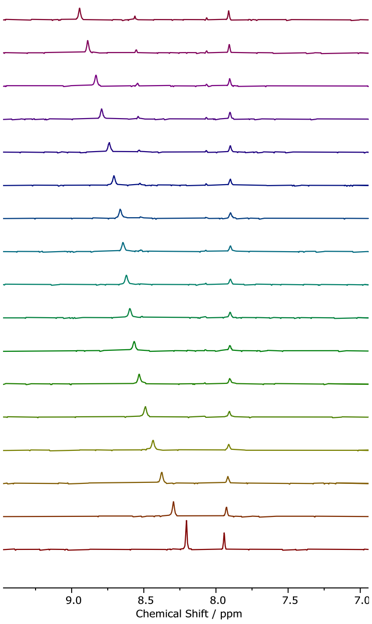

Figure S10. Stacked ^{1}H NMR TBACl titration of **2·Te^{2CF3}** (1mM).

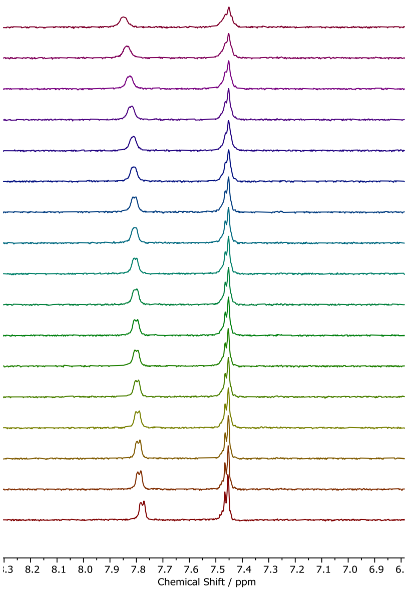

Figure S11. Stacked ^{1}H NMR TBACl titration of **2·Te^{Ph}** (1mM).

In the case of **1·Te^{2CF3}**, **1·Te^{Ph}**, **1·Se^{2CF3}**, **2·Se^{2CF3}**, **1·Se^{Ph}** and **2·Se^{Ph}** no chemical shift perturbations were observed after 10 equivalents of TBACl. A representative example is shown in for **2·Se^{2CF3}** below.

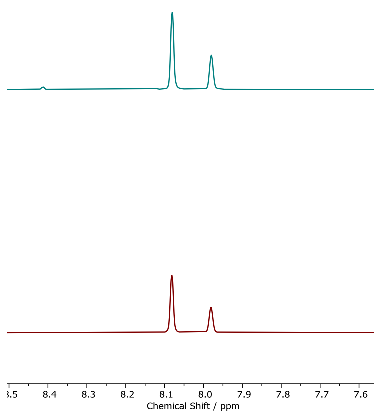

Figure S12. Stacked ^{1}H NMR TBACl titration of **2·Se^{2CF3}** (1mM).

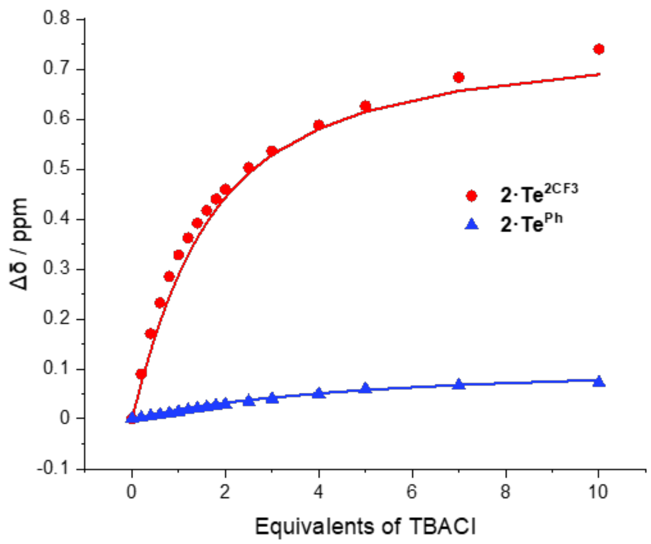

Figure S13. Chloride binding isotherm for **2·Te^{2CF3}** and **2·Te^{Ph}** where circles and triangles represent experimental data and the lines represent fitted data to a 1:1 binding isotherm.

Links to the fitting data for:

2·Te^{2CF3} http://app.supramolecular.org/bindfit/view/42f41203-0462-4eda-8a2e-dda40fface67

2·Te^{Ph} http://app.supramolecular.org/bindfit/view/9f08d026-d73e-4a04-865b-365710abe1ad

NMR Switching Studies

All switching studies were carried with at concentration of 5 mM of diaryl compound.

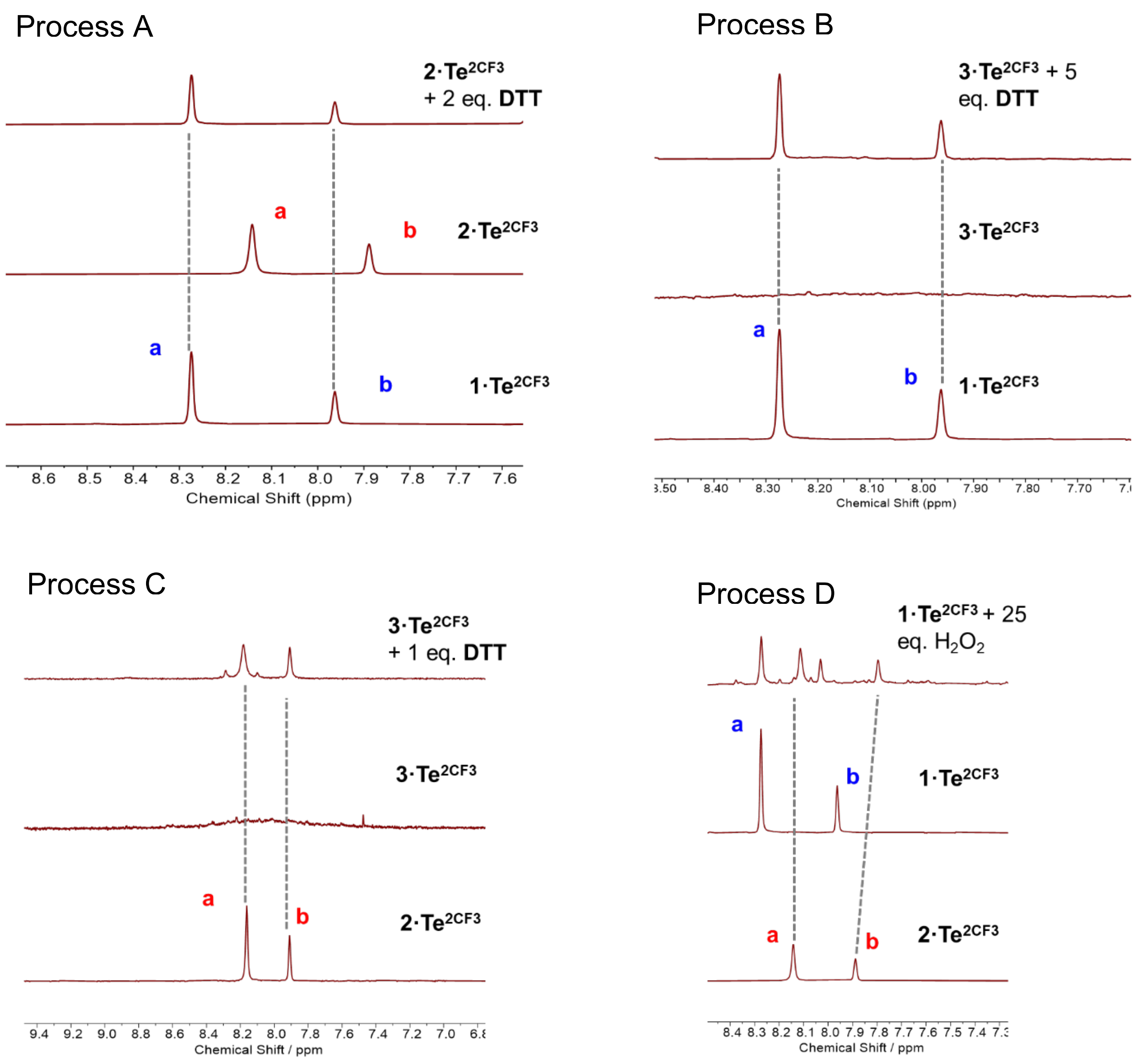

Figure S14. Truncated and stack ^{1}H NMR spectra of the representative switching process A, B, C and D, see scheme 2.

Synthesis and Characterisation

1·Te^{Ph}[1] and **2·Te^{Ph}**[2] were prepared according to literature procedures.

1·Se^{2CF3}[3] was prepared according to a literature procedure.

1·Se^{Ph} was purchased from Sigma-Aldrich.

2·Se^{Ph} was prepared according to a literature procedure.[2]

S1 was prepared according to a literature procedure.[4]

Tellurones, such as **3·Te^{2CF3}** and **3·Te^{Ph}**, present certain challenges with regards to their characterisation by NMR spectroscopy. The dynamic formation of higher aggregates and relatively poor solubility in most organic solvents causes significant broadening in ^{1}H, ^{13}C, ^{19}F and ^{125}Te NMR signals such that they are unobservable. It is important to recognise that this is a widely acknowledged characteristic of tellurones compounds. We present here the ^{1}H NMR spectra of **3·Te^{2CF3}** and **3·Te^{Ph}** to demonstrate this, but in all cases the $[M+H]^{+}$ cations were observable by ESI-MS.[5]

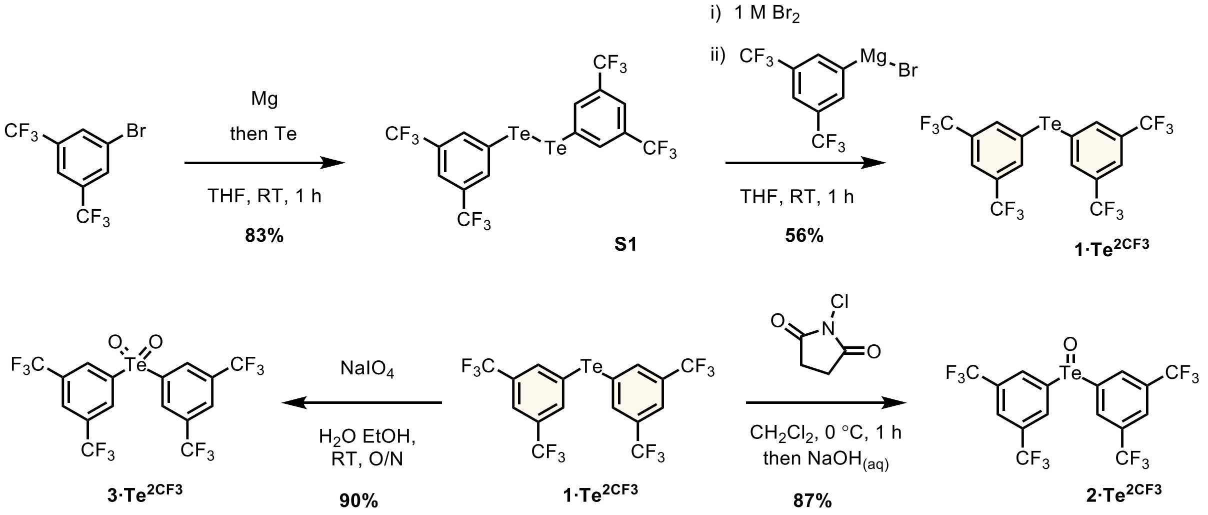

Scheme S1. Synthetic route for the tellurium-based receptors.

General procedure 1

The telluride (10.0 mmol) was dissolved in 20 mL of 1/1 (v/v) methanol/CH_2Cl_2, and the solution was cooled to 0 °C. NCS (1.41 g, 10.5 mmol) was added, and the resulting solution was stirred for 30 min at 0 °C. The reaction mixture was diluted with 20 mL of methylene chloride, and 30 mL of a 10% sodium hydroxide solution was added. After the mixture was stirred for 5 min, the organic phase was separated, dried over $MgSO_4$ and concentrated to dryness to afford the corresponding telluroxide as a white solid.

General procedure 2

The telluride (1.00 mmol) in ethanol (10 ml) was added a solution of sodium periodate (480.5 mg, 2.25 mmol) in water (10 ml). After being stirred at room temperature overnight, the mixture was diluted with water and partition with $CHCl_3$ (500 ml), the organic phase was collected and dried over $MgSO_4$ and concentrated to dryness affording the tellurone as a white solid.

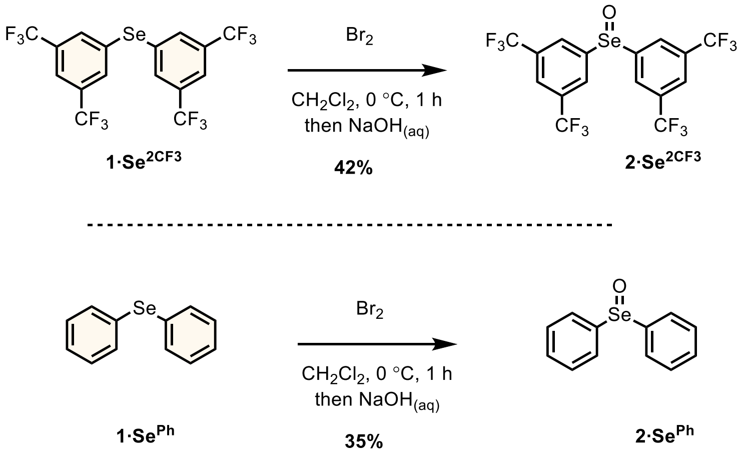

Scheme S2. Synthetic route for the selenium based receptors.

General procedure 3

The selenide (10.0 mmol) was dissolved in 20 mL of CH_2Cl_2, and the solution was cooled to 0 °C. Br_2 (10.5 mmol) was added, and the resulting solution was stirred for 30 min. The reaction mixture was diluted with 30 mL of a 10% sodium hydroxide solution was added. After the mixture was stirred for 5 min, the organic phase was separated, dried over $MgSO_4$ and concentrated to dryness to afford the corresponding selenoxide as a white solid.

1·Te^{2CF3}

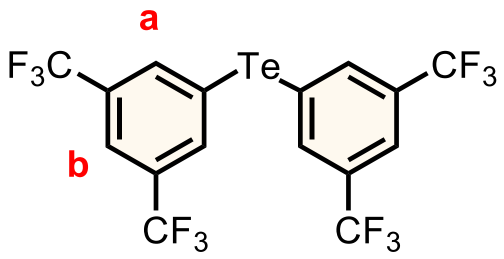

A solution of S1 (10 mmol) in anhydrous THF(50 ml) was cooled to 0°C. To which was added dropwise a 1 M Br_2 solution in ether (10 mmol) and allowed to stir for 5 minutes, after which time 3,5-Bis(trifluoromethyl)phenyl magnesium bromide (11 mmol) was added dropwise. After 30 minutes of stirring at room temperature the reaction mixture was quenched by the addition of MeOH (10 ml) and the mixture concentrated in vacuo. The crude mixture was portioned between brine (250 ml) and CH_2Cl_2 (250 ml), concentrated to dryness and purified by silica gel column chromatography eluting with (CH_2Cl_2:hexane, 9:1, v/v) to afford **1·Te**^{2CF3} as orange solid (56%).

^{1}**H NMR** (400 MHz, Acetone-d_6) δ 8.40 (d, *J* = 1.8 Hz, 2H_a), 8.03 (t, *J* = 1.8 Hz, 1H_b).

^{125}**Te NMR** (126 MHz, Acetone-d_6) δ 820.71.

^{13}**C NMR** (101 MHz, Acetone-d_6) δ 139.02 (d, *J* = 3.9 Hz), 132.72 (q, *J* = 33.2 Hz), 123.96(q, *J* = 273.71 Hz) 123.05 (p, *J* = 4.0 Hz), 118.72.

^{19}**F NMR** (377 MHz, Acetone-d_6) δ -63.59.

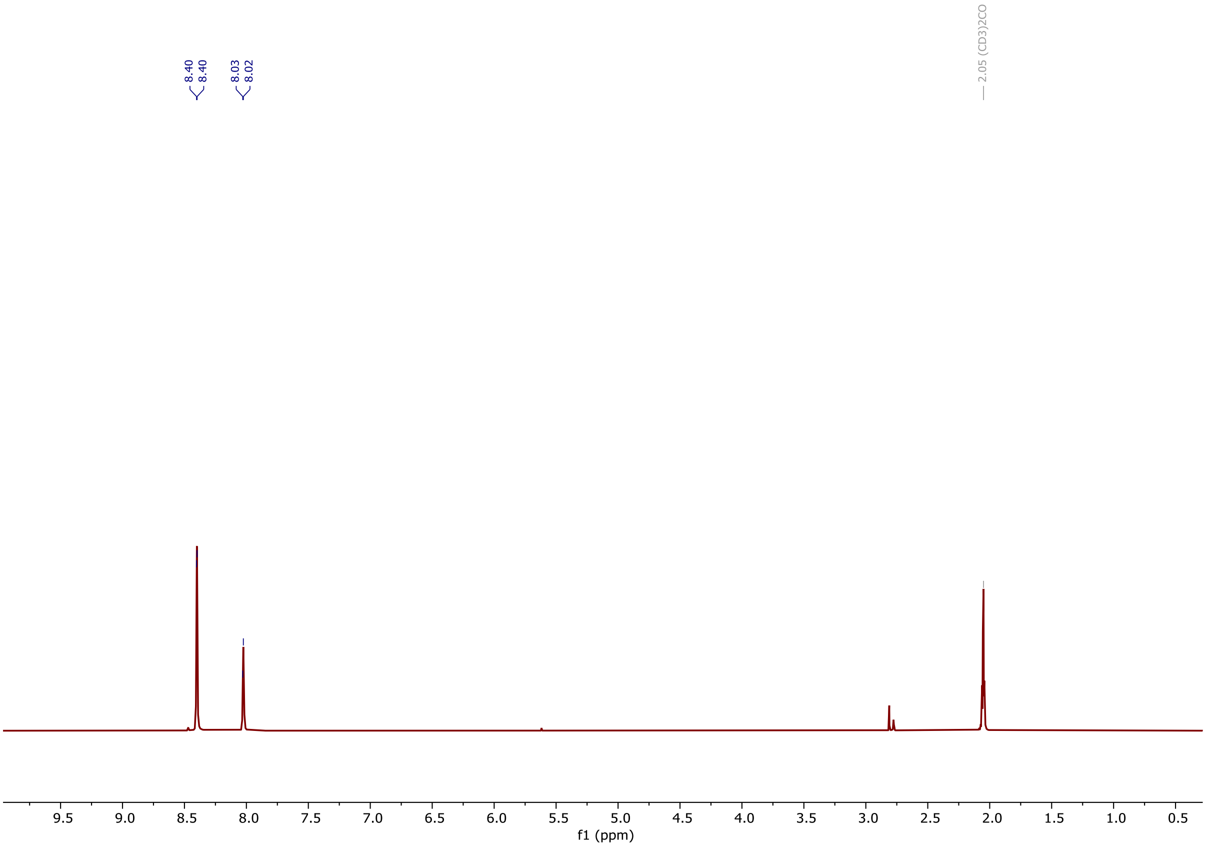

Figure S15. ^{1}H NMR Spectrum of **1·Te^{2CF3}** (Acetone-d_6, 400 MHz, 298K).

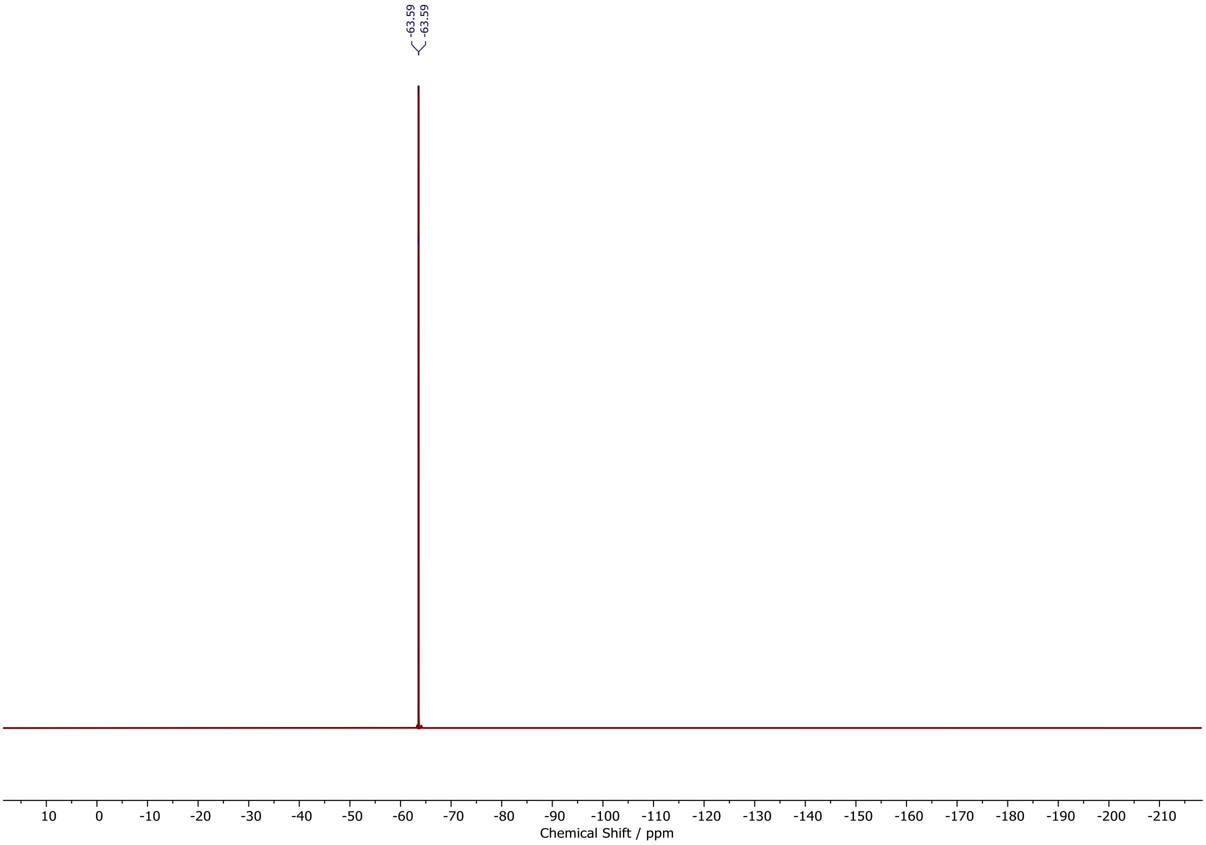

Figure S16. ^{19}F NMR Spectrum of **1·Te^{2CF3}** (Acetone-d$_6$, 377 MHz, 298K).

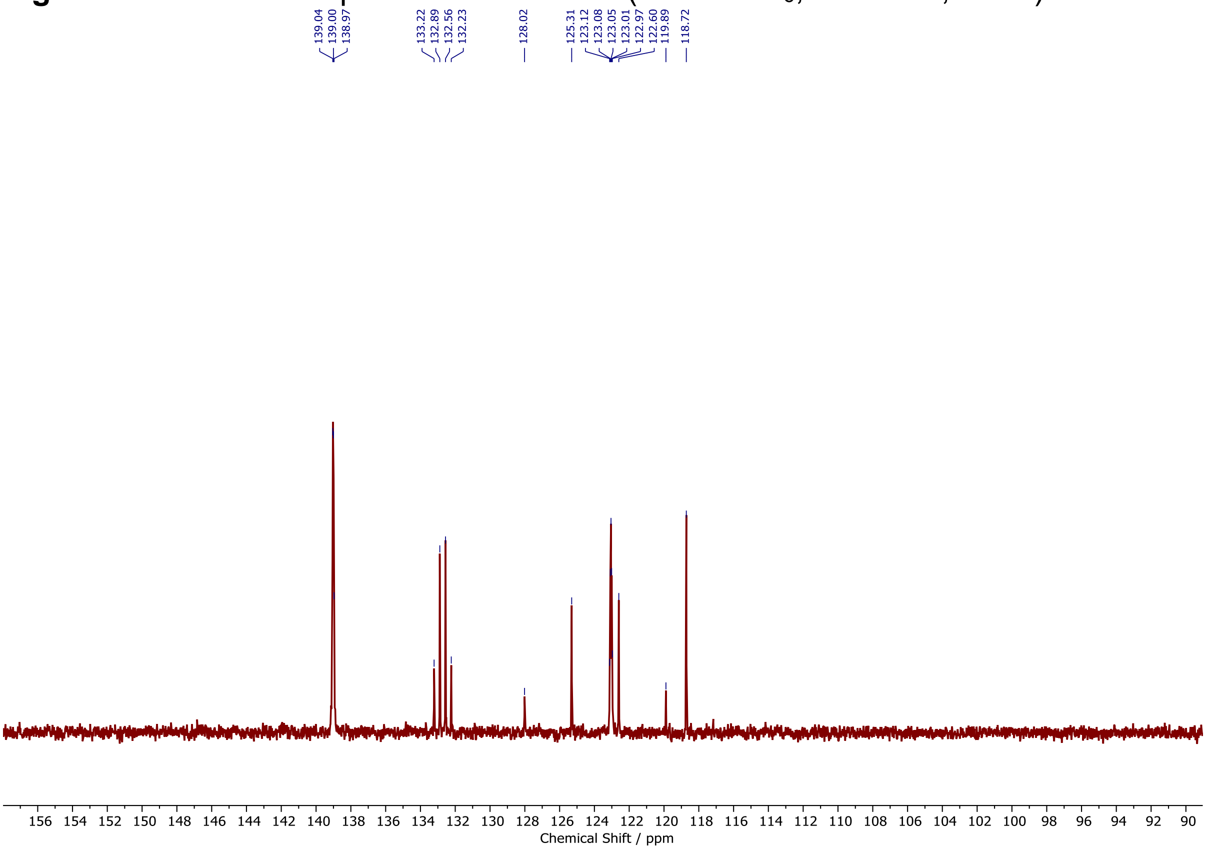

Figure S17. ^{13}C NMR Spectrum of **1·Te^{2CF3}** (Acetone-d$_6$, 101 MHz, 298K).

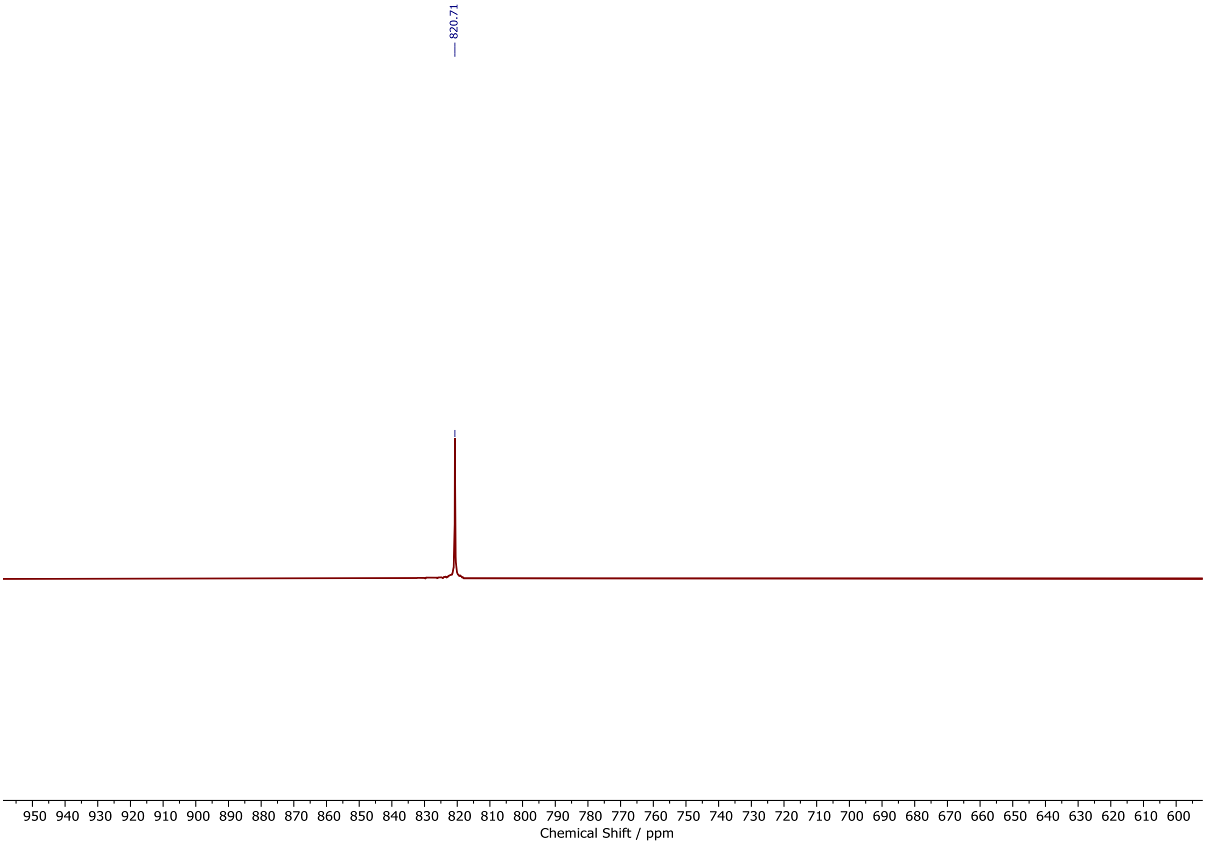

Figure S18. ^{125}Te NMR Spectrum of **1·Te^{2CF3}** (Acetone-d$_6$, 126 MHz, 298K).

2·Te^{2CF3}

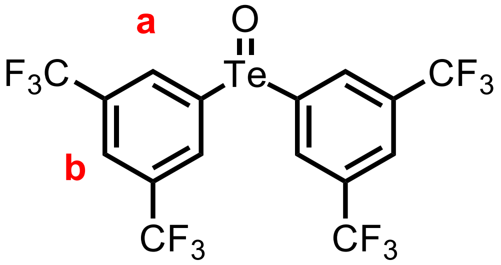

Synthesised using General procedure 1

^{1}H NMR (400 MHz, Acetone-d_6) δ 8.31 (d, *J* = 1.7 Hz, 2H_a), 7.93 (t, *J* = 1.7 Hz, 1H_b).

^{125}Te NMR (126 MHz, Acetone-d_6) δ 1095.84.

^{13}C NMR (101 MHz, Acetone-d_6) δ 145.82, 131.68, 131.33 (q, *J* = 32 Hz), 123.9 (m), 123.19 (q, *J* = 247 Hz).

^{19}F NMR (377 MHz, Acetone-d_6) δ -63.58.

HRMS (ESI+ve) m/z: 572.9389 ($[M+H]^+$, $C_{16}H_7F_{12}O^{130}Te$ requires 572.9368).

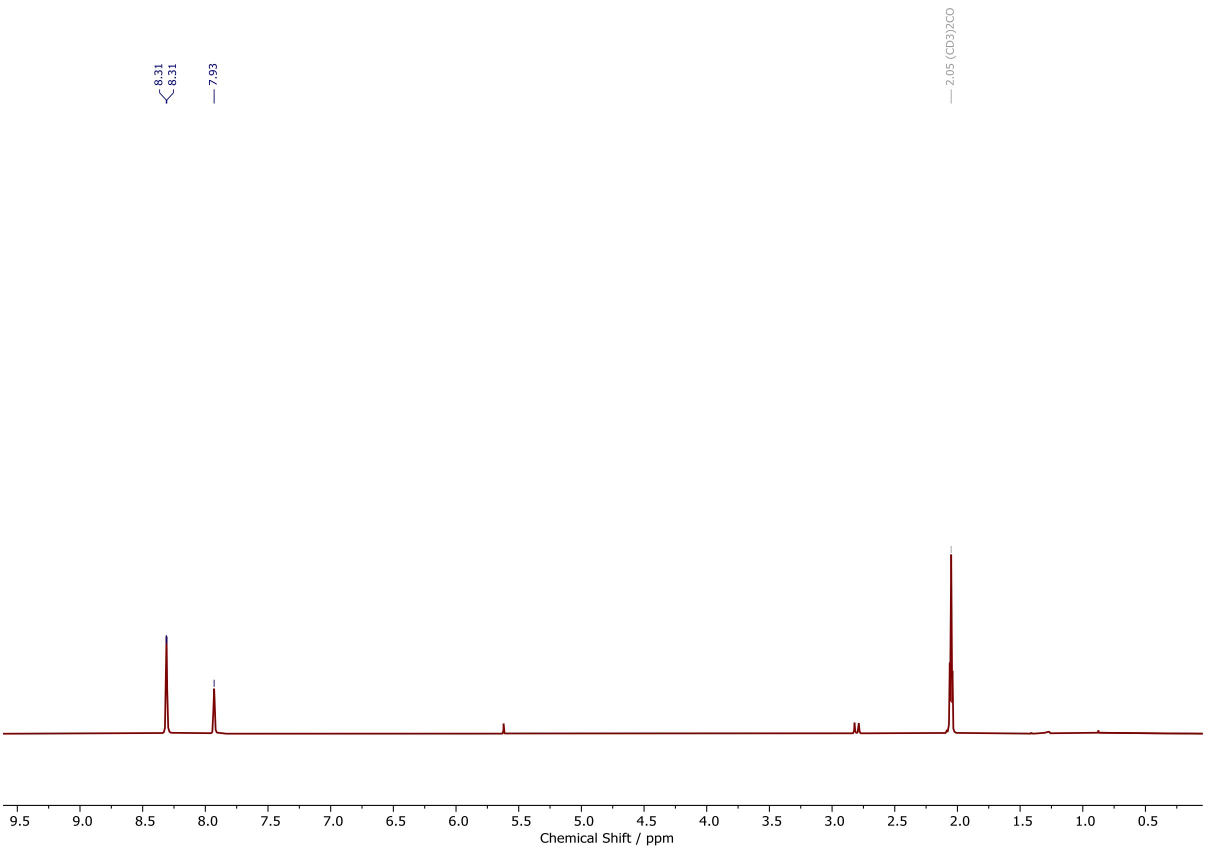

Figure S19. ^{1}H NMR Spectrum of **2·Te^{2CF3}** (Acetone-d$_6$, 400 MHz, 298K).

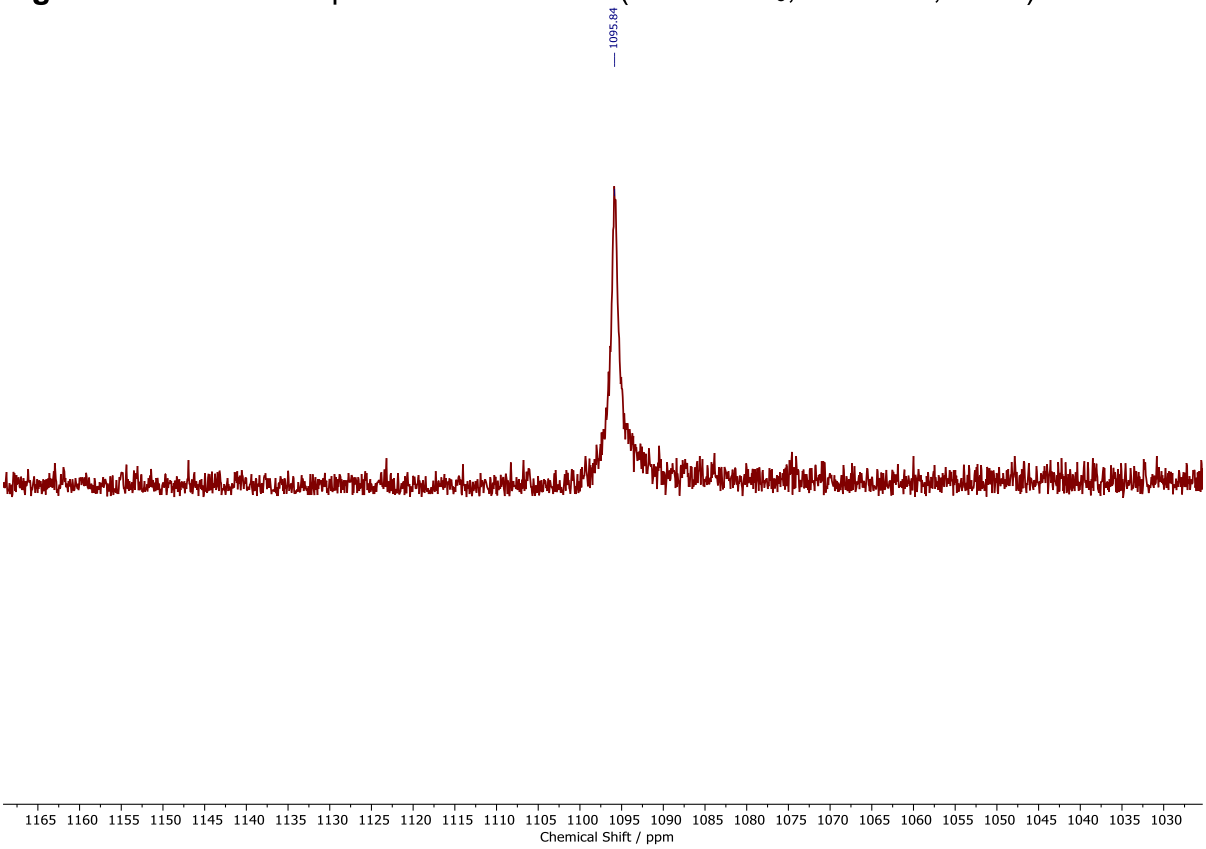

Figure S20. ^{125}Te NMR Spectrum of **2·Te^{2CF3}** (Acetone-d$_6$, 126 MHz, 298K).

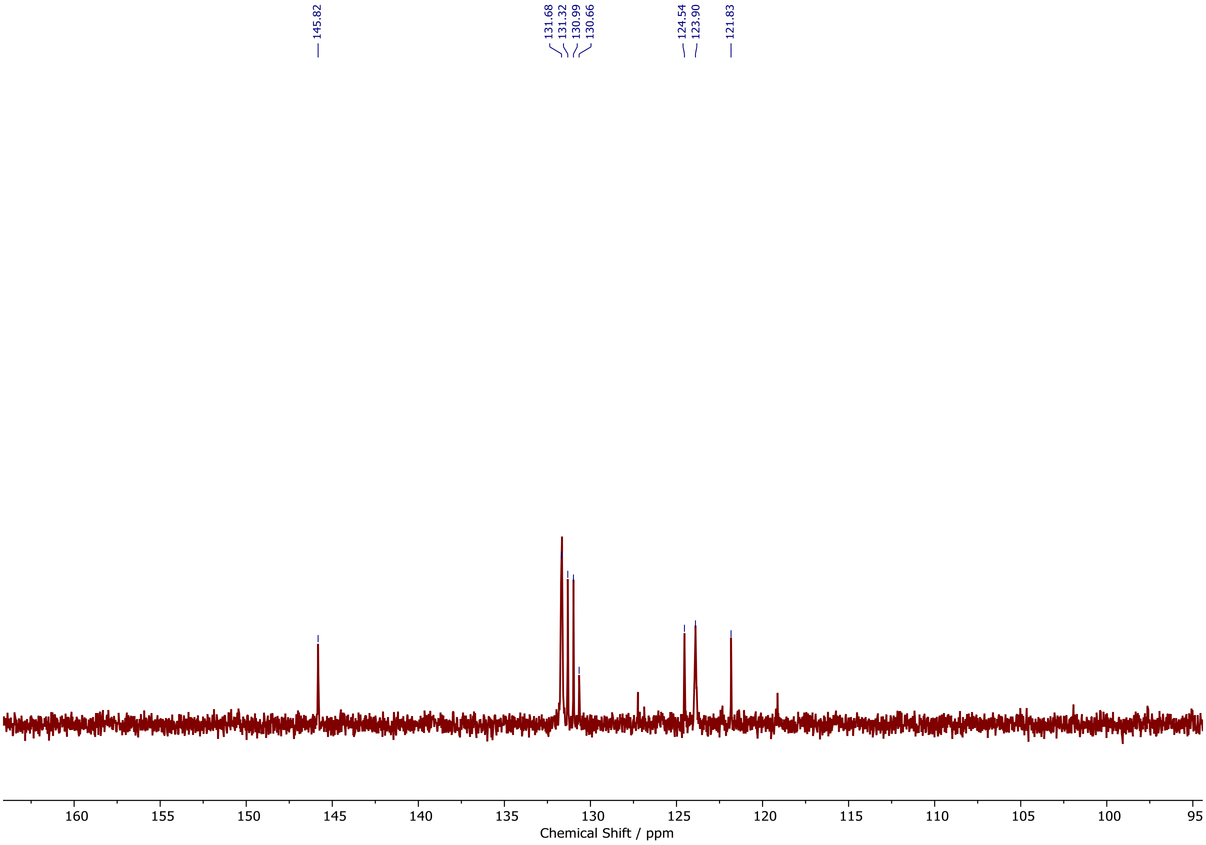

Figure S21. ^{13}C NMR Spectrum of **2·Te^{2CF3}** (Acetone-d_6, 101 MHz, 298K).

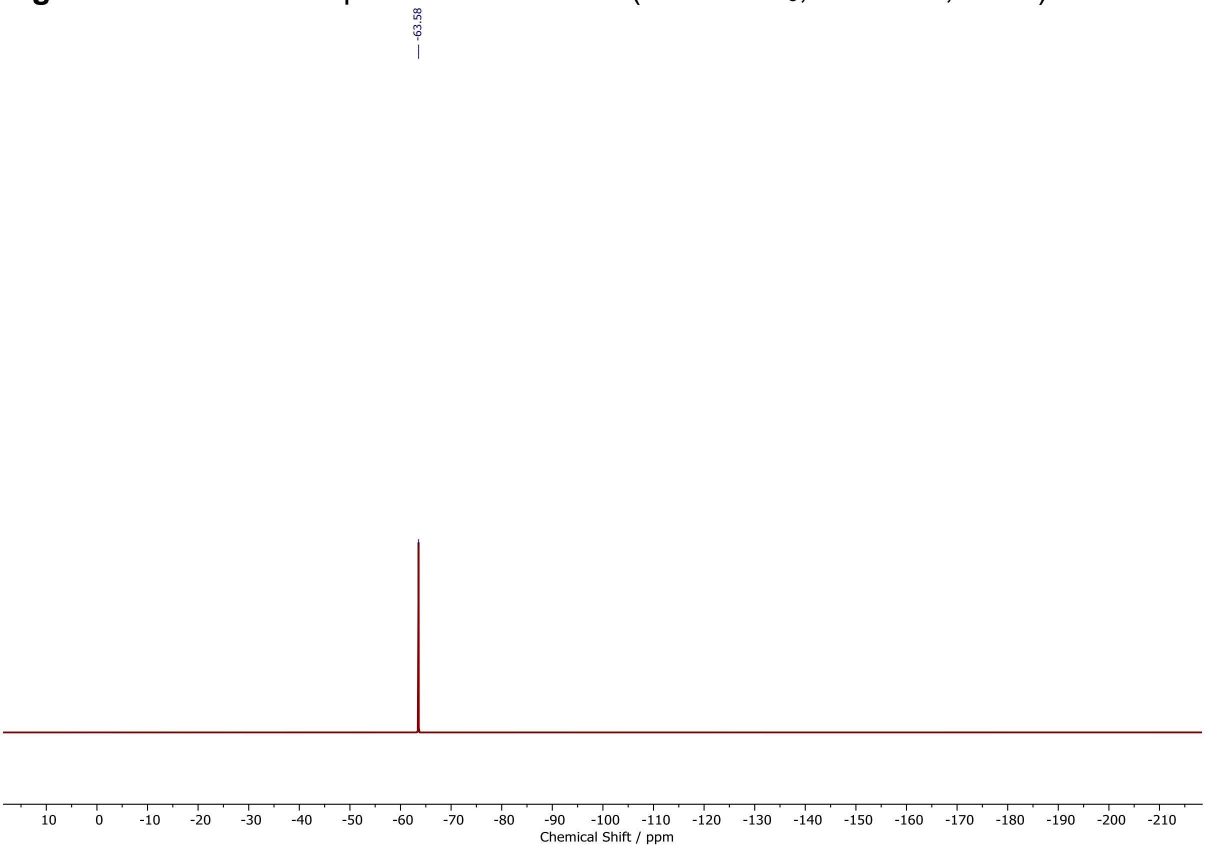

Figure S22. ^{19}F NMR Spectrum of **2·Te^{2CF3}** (Acetone-d_6, 377 MHz, 298K).

3·Te^2CF3^

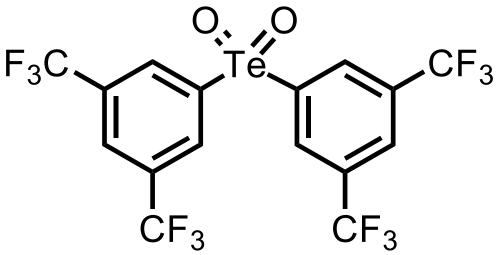

Synthesised using General procedure 2

HRMS (ESI+ve) m/z: 588.9364 ($[M+H]^+$, $C_{16}H_7F_{12}O_2{}^{130}Te$ requires 588.9317).

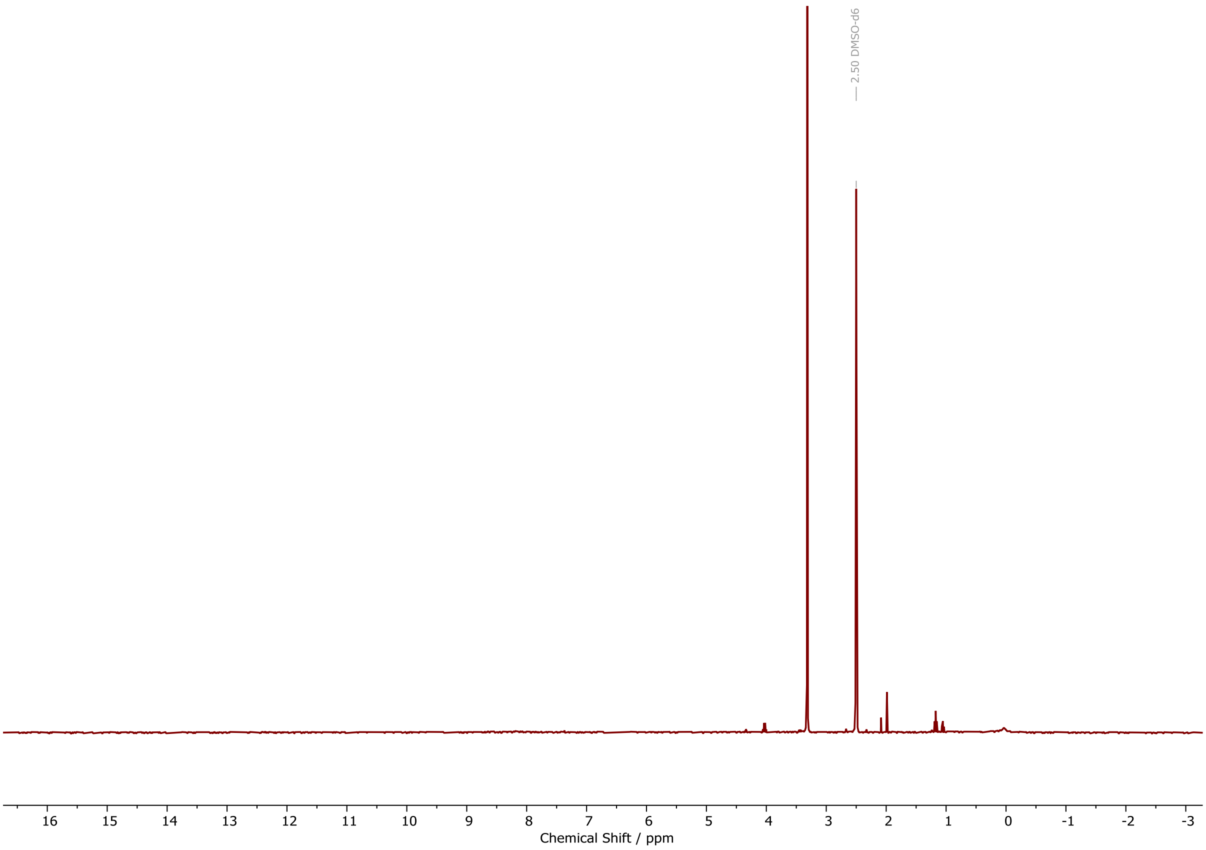

Figure S23. ^1H NMR Spectrum of **3·Te^2CF3^** (DMSO-d$_6$ 400 MHz, 298K).

3·Te^{Ph}

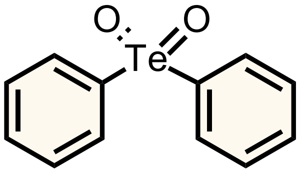

Synthesised using General procedure 2

HRMS (ESI+ve) m/z: 316.9857 ($[M+H]^+$, $C_{12}H_{11}O_2{}^{130}Te$ requires 316.9821).

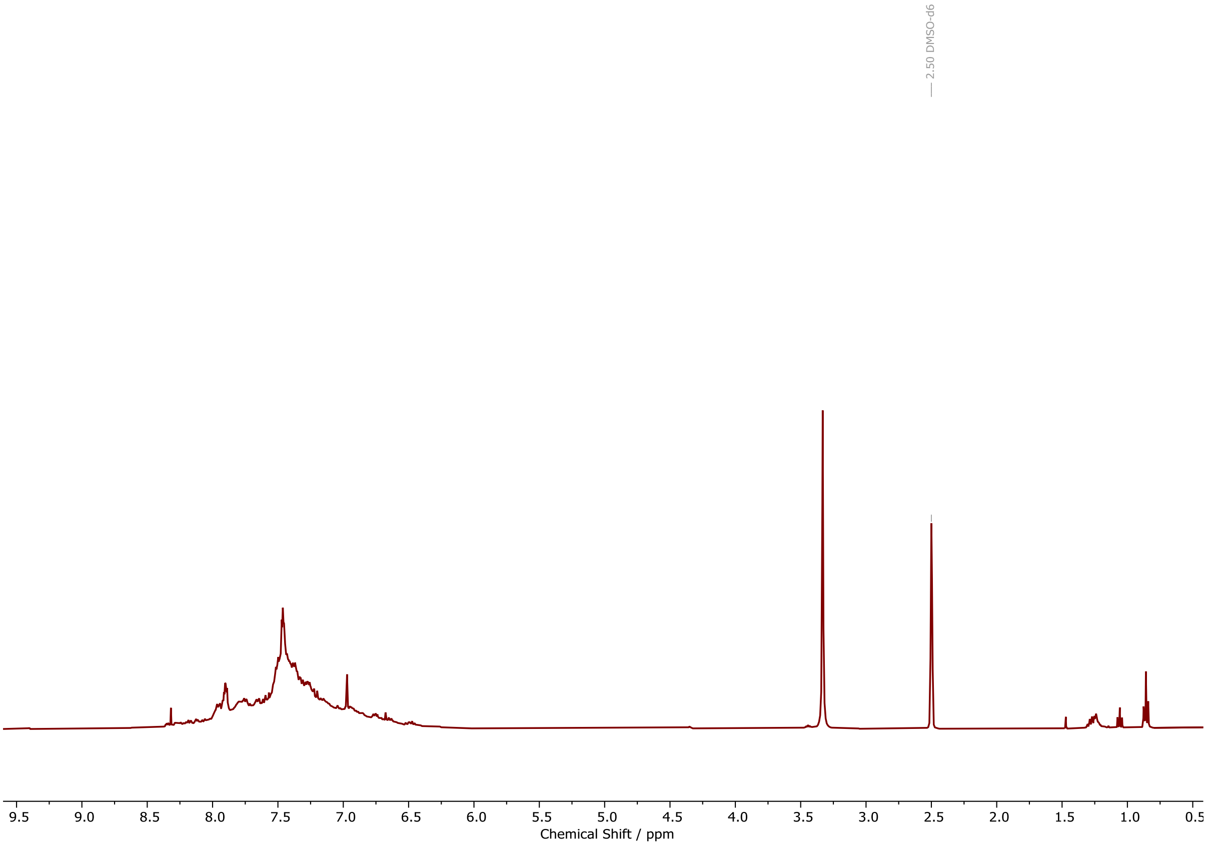

Figure S24. ^1H NMR Spectrum of **3·Te^{Ph}** (DMSO-d_6 400 MHz, 298K).

1·Se[2CF3]

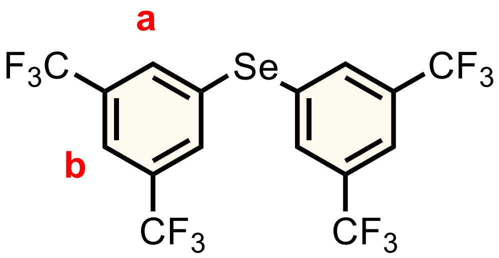

Isolated as white solid (38%).

^{1}H NMR (400 MHz, $CDCl_3$) δ 7.91 (d, *J* = 1.7 Hz, $2H_a$), 7.89 – 7.80 (m, $1H_b$).

^{13}C NMR (101 MHz, $CDCl_3$) δ 133.13 (q, *J* = 34 Hz), 132.99 (m), 132.23, 123.13 (q, *J* = 273 Hz), 122.88 – 122.26 (m).

^{19}F NMR (377 MHz, $CDCl_3$) δ -63.59.

^{77}Se NMR (95 MHz, $CDCl_3$) δ 416.37.

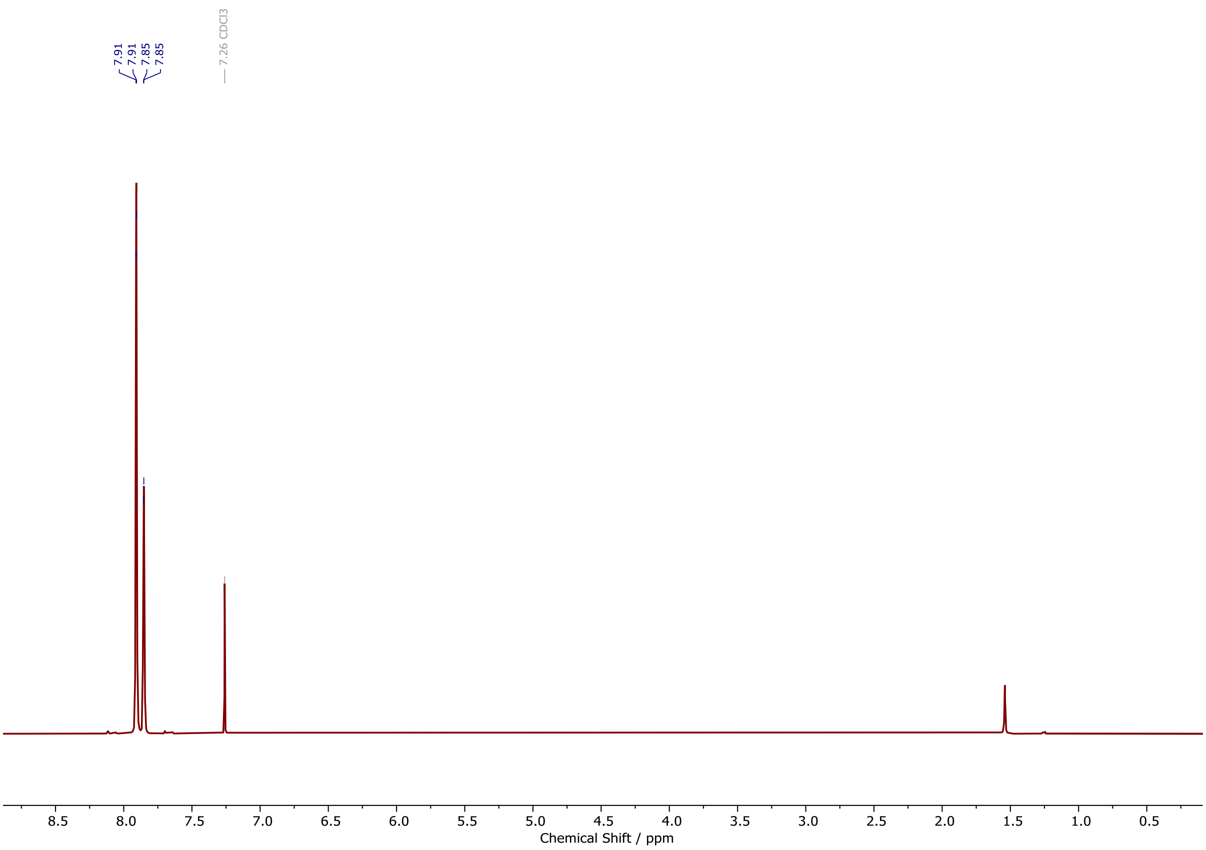

Figure S25. ^{1}H NMR Spectrum of **1·Se^{2CF3}** ($CDCl_3$, 400 MHz, 298K).

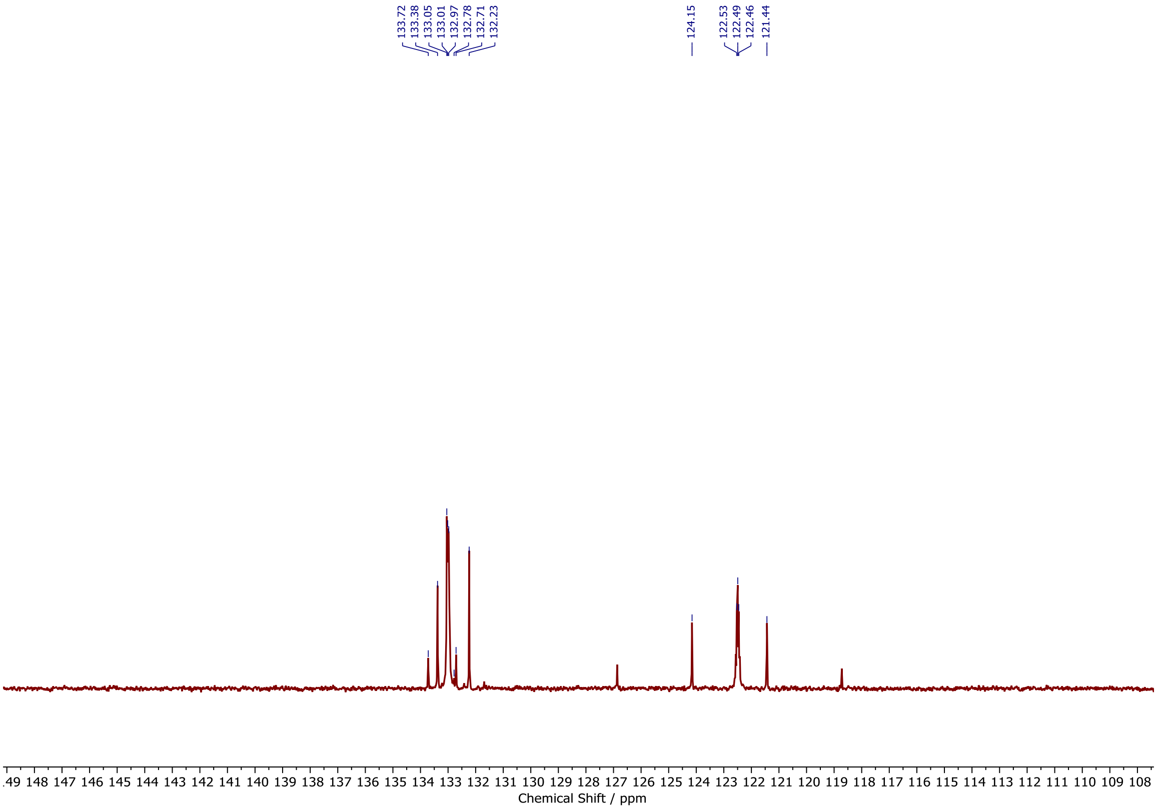

Figure S26. ^{13}C NMR Spectrum of **1·Se^{2CF3}** ($CDCl_3$, 101 MHz, 298K).

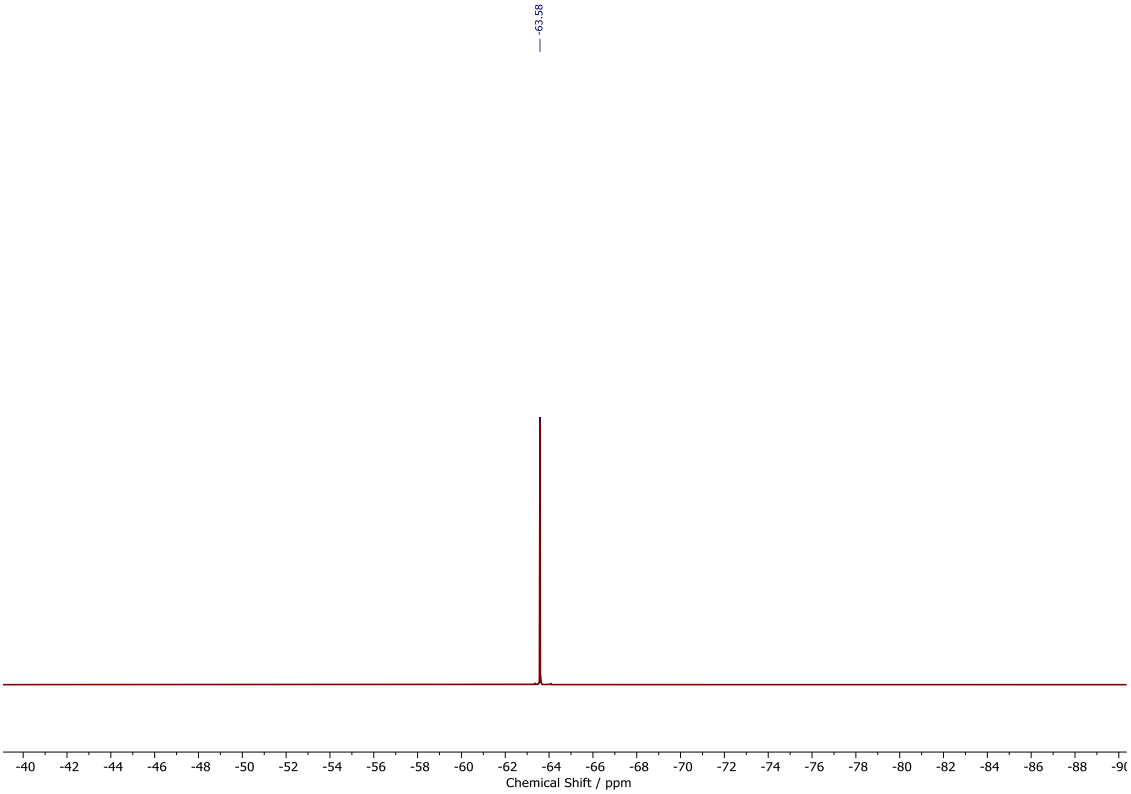

Figure S27. ^{19}F NMR Spectrum of **1·Se^{2CF3}** ($CDCl_3$, 377 MHz, 298K).

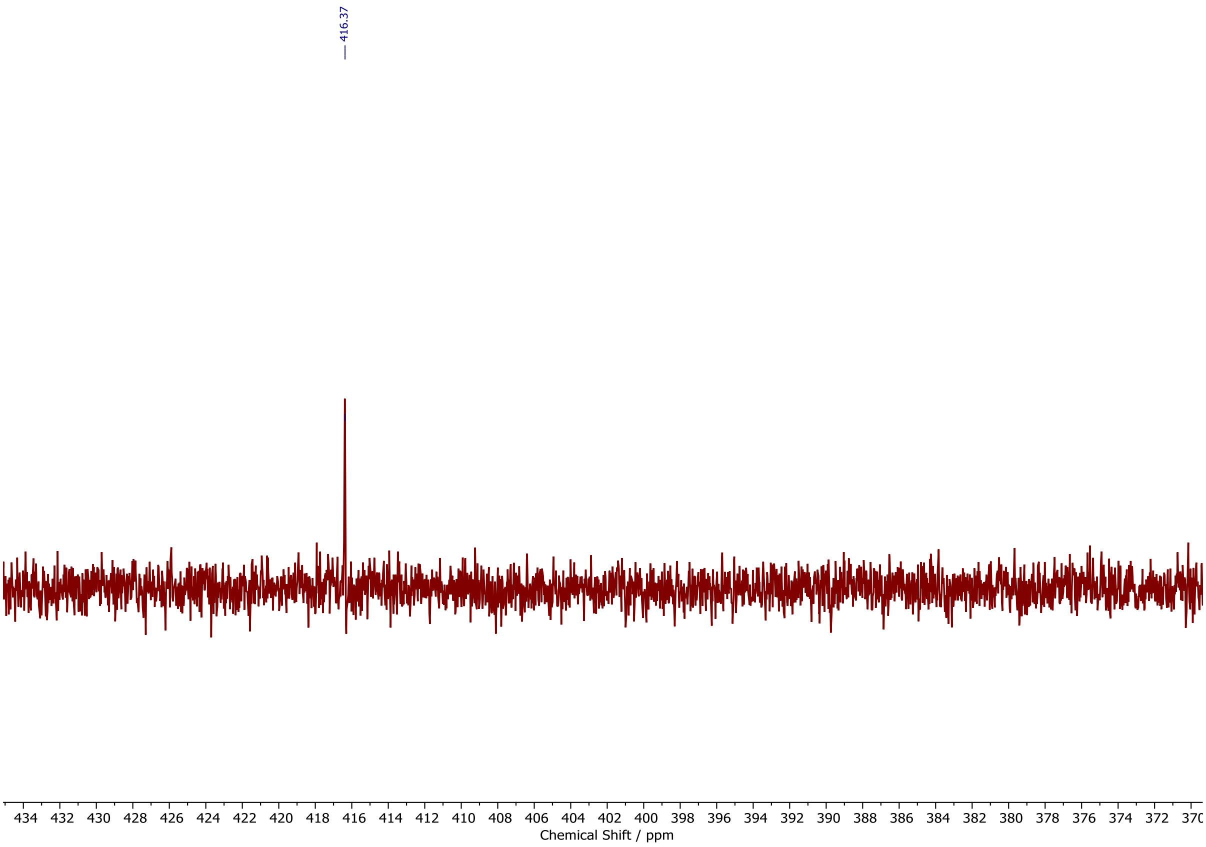

Figure S28. ^{77}Se NMR Spectrum of **1·Se^{2CF3}** ($CDCl_3$, 95 MHz, 298K).

2·Se^2CF3

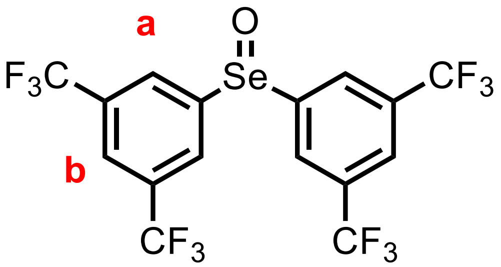

Synthesised using General procedure 3

^{1}H NMR (400 MHz, CD_3CN) δ 8.65 – 8.35 (m, $2H_a$), 8.12 (t, *J* = 1.6 Hz, $1H_b$).

^{13}C NMR (101 MHz, CD_3CN) δ 157.82, 143.68, 143.16 (q, *J* = 33 Hz) 135.90, 135.19 (q, *J* = 224 Hz).

^{19}F NMR (377 MHz, CD_3CN) δ -68.58.

^{77}Se NMR (95 MHz, CD_3CN) δ 488.83 (d, *J* = 4.2 Hz).

HRMS (ESI+ve) m/z: 522.9438 ($[M+H]^+$, $C_{16}H_7F_{12}O^{80}Se$ requires 522.9471).

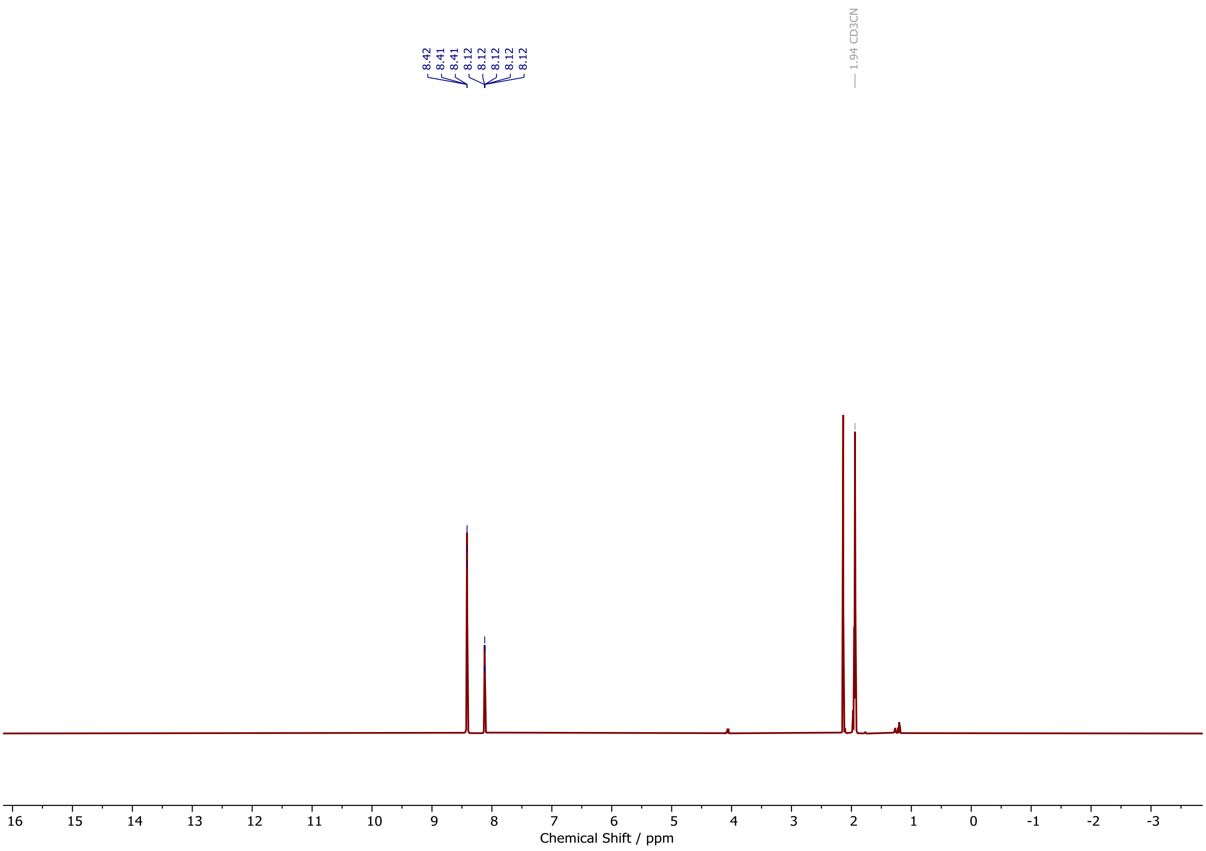

Figure S29. ^{1}H NMR Spectrum of **2·Se$^{\mathbf{2CF3}}$** (CD_3CN, 400 MHz, 298K).

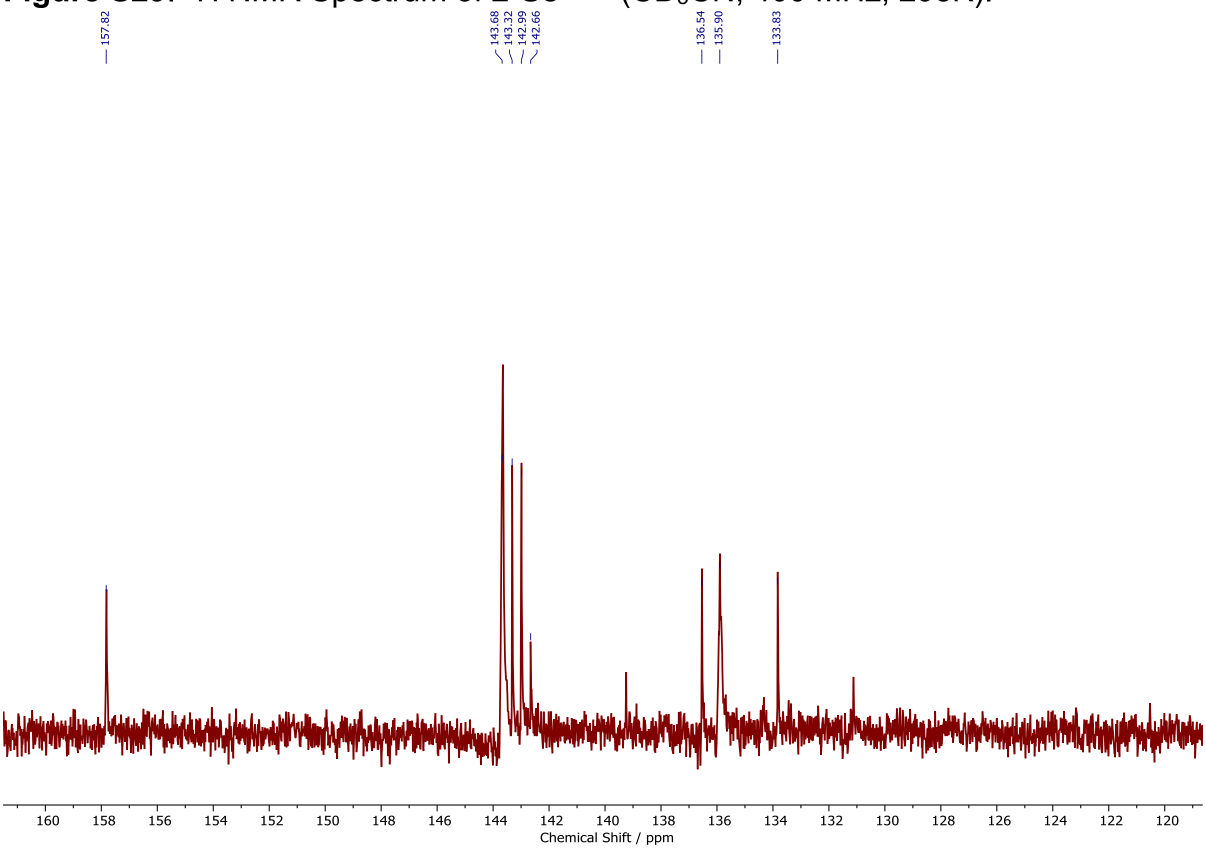

Figure S30. ^{13}C NMR Spectrum of **2·Se$^{\mathbf{2CF3}}$** (CD_3CN, 101 MHz, 298K).

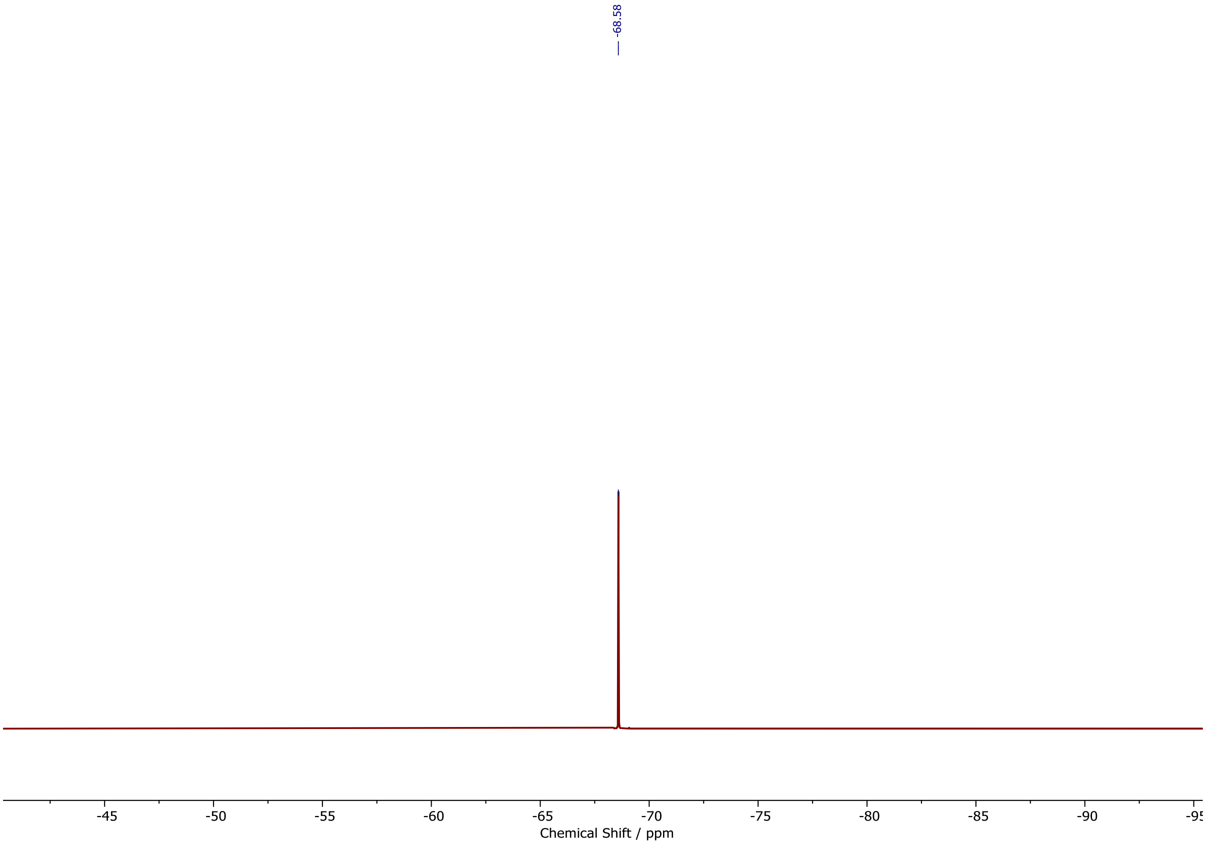

Figure S31. ^{19}F NMR Spectrum of **2·Se^{2CF3}** (CD_3CN, 377 MHz, 298K).

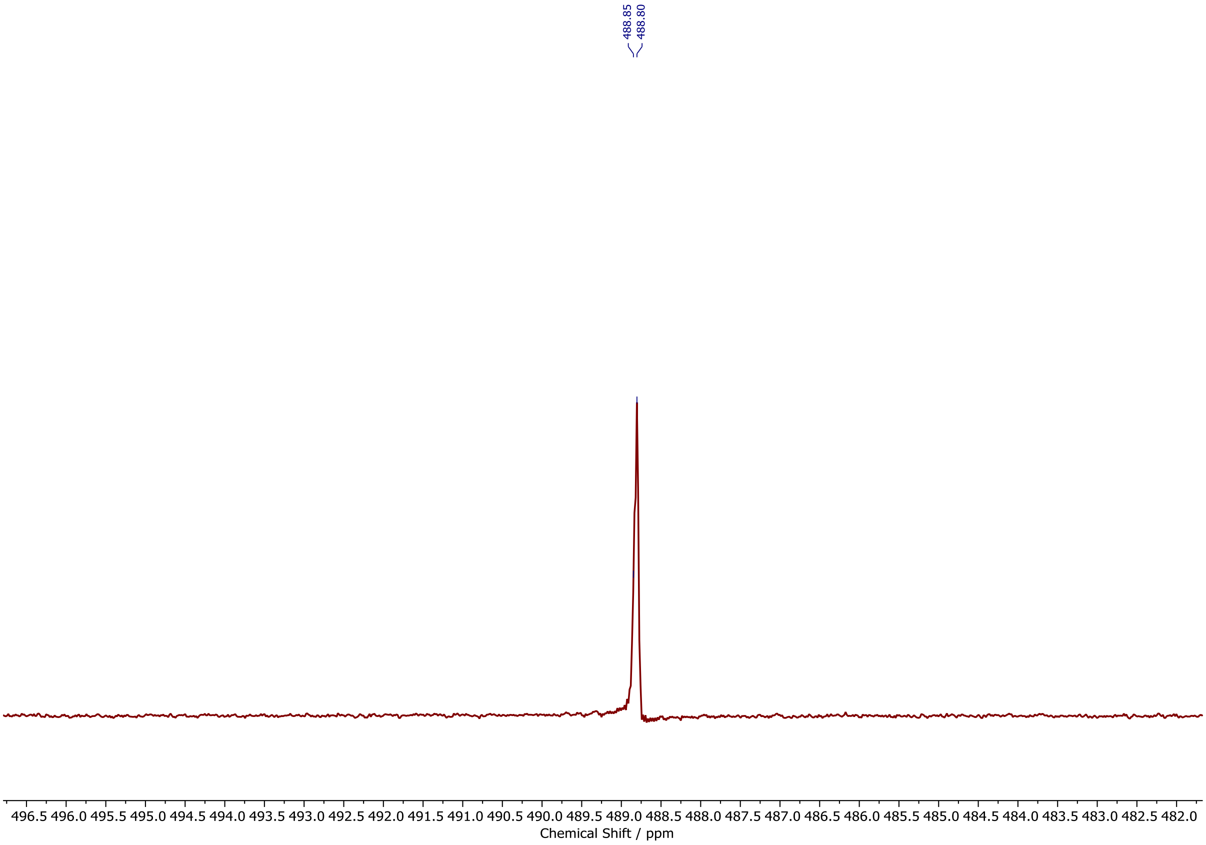

Figure S32. ^{77}Se NMR Spectrum of **2·Se^{2CF3}** (CD_3CN, 95 MHz, 298K).

Single Crystal X-ray diffraction experiments

Crystal Structure Determination Deposition Numbers 2194578 (for **2·Te^{2CF3}**) and 2194798 (for **2·Se^{2CF3}**) contains the supplementary crystallographic data for this paper. These data are provided free of charge by the joint Cambridge Crystallographic Data Centre and Fachinformationszentrum Karlsruhe Access Structures service www.ccdc.cam.ac.uk/structures. Single-crystal X-ray diffraction intensities for **2·Te^{2CF3}** and **2·Se^{2CF3}** were collected at 150 K on Oxford Diffraction/Agilent SuperNovae diffractometers with Cu-Kα (λ = 1.54184 Å) radiation equipped with a nitrogen gas Oxford Cryosystems Cryostream unit A suitable crystal was chosen and mounted on a 200 μm MiTeGen loop using perfluoropolyether oil. The CrysAlisPro software was used for data collection and integration. Structure **2·Te^{2CF3}** was solved using SuperFlip and refined using full-matrix least-squares refinement within the CRYSTALS suite. Structure **2·Se^{2CF3}** was further refined using full-matrix least-squares refinement using Shelxl-2014. All non-hydrogen atoms were refined anisotropically. The hydrogen atoms were positioned at geometrically sensible positions and refined using a riding model.

Table S1. Crystallographic table for **2·Te^{2CF3}**.

**Compound**	**2·Te^{2CF3}**
**Formula**	C_{23} H_{12} F_{24} O_2 Te_2 [+solvent]
**Formula Weight**	1139.60
**a (Å)**	14.7746(2)
**b (Å)**	15.5350(2)
**c (Å)**	20.1624(3)
α **(°)**	74.3377(11)
β **(°)**	84.7562(1)
γ **(°)**	68.3659(11)
**Unit cell volume (Å^3)**	4141.85(11)
**Crystal system**	Triclinic
**Space group**	P -1
**Z**	4
**Temperature (K)**	150 K
**Radiation Type**	Copper
**λ (Å)**	1.54180
**Reflections (all)**	112512
**Reflections (unique)**	17183
**R_{int}**	0.034
**$R[I > 2\sigma(I)]$**	0.0587
**$wR(F^2)$ (all)**	0.1572
**S**	1.000

The compound crystallised with disordered solvent, which was treated using PLATON SQUEEZE. Disorder can also be observed for some of the CF_3 groups, which has been modelled. However, some of the ADPs for the CF_3 groups remain enlarged due to the disorder present.

Table S2. Crystallographic table for **2·Se^{2CF3}**.

**Compound**	**2·Se^{2CF3}**
**Formula**	C_{16} H_6 F_{12} O Se
**Formula Weight**	521.17
**a (Å)**	9.1317(3)
**b (Å)**	16.9127(7)
**c (Å)**	23.1422(13)
α **(°)**	90°
β **(°)**	95.175(4)°
γ **(°)**	90°
**Unit cell volume (Å^3)**	3559.6(3)
**Crystal system**	Monoclinic
**Space group**	P21
**Z**	8
**Temperature (K)**	150 K
**Radiation Type**	Copper
**λ (Å)**	1.54180
**Reflections (all)**	42344
**Reflections (unique)**	14631
**R_{int}**	0.102
**R[I > 2σ(I)]**	0.1346
**wR(F^2) (all)**	0.3978
**S**	1.492

Disorders were observed for Se atoms and were modelled into occupancy of 0.81 and 0.19, respectively. The disordered parts of Se atoms caused some short inter D...A contact alerts reported by Checkcif.

References

[1] S. Kumar, L. Engman, *J. Org. Chem.* **2006**, *71*, 5400–5403.
[2] M. R. Detty, *J. Org. Chem.* **1980**, *45*, 274–279.
[3] M. A. Rizvi, M. Zaki, Mohd. Afzal, M. Mane, M. Kumar, B. A. Shah, S. Srivastav, S. Srikrishna, G. M. Peerzada, S. Tabassum, *Eur. J. Med. Chem.* **2015**, *90*, 876–888.
[4] A. Docker, C. H. Guthrie, H. Kuhn, P. D. Beer, *Angew. Chem. Int. Ed.* **2021**, *60*, 21973–21978.
[5] K. Srivastava, A. Panda, S. Sharma, H. B. Singh, *J. Organomet. Chem.* **2018**, *861*, 174–206.
